# Inhibition of HSP27 alone or in combination with pAKT inhibition as therapeutic approaches to target SPARC-induced glioma cell survival

**DOI:** 10.1186/1476-4598-11-20

**Published:** 2012-04-05

**Authors:** Chad R Schultz, William A Golembieski, Daniel A King, Stephen L Brown, Chaya Brodie, Sandra A Rempel

**Affiliations:** 1 The Barbara Jane Levy Laboratory of Molecular Neuro-Oncology, Henry Ford Hospital, 2799 West Grand Blvd., Detroit, MI 48202, USA; 2 The William & Karen Davidson Laboratory of Cell Signaling and Tumorigenesis, Hermelin Brain Tumor Center, Department of Neurosurgery, Henry Ford Hospital, 2799 West Grand Blvd., Detroit, MI 48202, USA; 3 Department of Radiation Oncology, Henry Ford Hospital, 2799 West Grand Blvd., Detroit, MI 48202, USA

**Keywords:** Glioma, SPARC, HSP27, AKT, Tumor cell survival, Apoptosis, Autophagy, Temozolomide

## Abstract

**Background:**

The current treatment regimen for glioma patients is surgery, followed by radiation therapy plus temozolomide (TMZ), followed by 6 months of adjuvant TMZ. Despite this aggressive treatment regimen, the overall survival of all surgically treated GBM patients remains dismal, and additional or different therapies are required. Depending on the cancer type, SPARC has been proposed both as a therapeutic target and as a therapeutic agent. In glioma, SPARC promotes invasion via upregulation of the p38 MAPK/MAPKAPK2/HSP27 signaling pathway, and promotes tumor cell survival by upregulating pAKT. As HSP27 and AKT interact to regulate the activity of each other, we determined whether inhibition of HSP27 was better than targeting SPARC as a therapeutic approach to inhibit both SPARC-induced glioma cell invasion and survival.

**Results:**

Our studies found the following. **1) **SPARC increases the expression of tumor cell pro-survival and pro-death protein signaling in balance, and, as a net result, tumor cell survival remains unchanged. **2) **Suppressing SPARC increases tumor cell survival, indicating it is not a good therapeutic target. **3) **Suppressing HSP27 decreases tumor cell survival in all gliomas, but is more effective in SPARC-expressing tumor cells due to the removal of HSP27 inhibition of SPARC-induced pro-apoptotic signaling. **4) **Suppressing total AKT1/2 paradoxically enhanced tumor cell survival, indicating that AKT1 or 2 are poor therapeutic targets. **5) **However, inhibiting pAKT suppresses tumor cell survival. **6) **Inhibiting both HSP27 and pAKT synergistically decreases tumor cell survival. **7) **There appears to be a complex feedback system between SPARC, HSP27, and AKT. **8) **This interaction is likely influenced by PTEN status. With respect to chemosensitization, we found the following. **1) **SPARC enhances pro-apoptotic signaling in cells exposed to TMZ. **2) **Despite this enhanced signaling, SPARC protects cells against TMZ. **3) **This protection can be reduced by inhibiting pAKT. **4) **Combined inhibition of HSP27 and pAKT is more effective than TMZ treatment alone.

**Conclusions:**

We conclude that inhibition of HSP27 alone, or in combination with pAKT inhibitor IV, may be an effective therapeutic approach to inhibit SPARC-induced glioma cell invasion and survival in SPARC-positive/PTEN-wildtype and SPARC-positive/PTEN-null tumors, respectively.

## Background

Glioblastomas (GBMs) are the most malignant and heterogeneous human brain tumors [1]. Approximately 90%-95% of GBMs develop rapidly without evidence of lower grade precursor tumors. These are designated as primary or "*de novo*" tumors [2]. The remaining 5%-10% develop through progressive changes from low-grade diffuse astrocytoma and/or anaplastic astrocytoma and are designated as secondary GBMs [2]. Sequencing, copy number analysis, and expression profiles have better delineated the genetic alterations present in the tumors, and permit an analysis of major signaling pathways disrupted in primary GBMs [3,4]. Three major signaling pathways are commonly disrupted. EGFR and PTEN mutation/deletion/methylation are the most common in the RTK/RAS/PI3K signaling pathway, p53 mutation/deletion in the p53 pathway, and CDKN2B mutation/deletion in the RB pathway. Fewer secondary GBMs have been analyzed as comprehensively; however, they appear to share some of the same genetic defects as primary GBMs. One exception is IDH1, which is highly, but not exclusively, mutated in secondary GBMs [4]. Gene expression profiling and integrated genomic analyses of a large number of tumors [5,6] have been pivotal in defining subtypes of GBM that differ in their genetic mutations and in their response to therapy [6].

The standard of care for newly diagnosed GBM patients has been impacted by such analyses. Presently, treatment includes surgery followed by treatment with temozolomide (TMZ) plus radiotherapy followed by 6 months of adjuvant TMZ treatment [7,8]. This treatment is most successful against tumors having a methylated O^6^-methylguanine-DNA-methyltransferase (MGMT) gene. The methylation silences the gene thereby inhibiting the expression of an enzyme that repairs TMZ-induced DNA damage, permitting increased tumor cell death. This treatment regimen increases progression-free survival at six months and overall survival time to 14.6 months for selected patients [9]; however, the median overall survival for all patients operated for primary GBM ranges from 9.9 to 10.2 months [10]. Therefore, different or additional adjuvant therapies are required.

Secreted protein acidic and rich in cysteine (SPARC [11]), also known as osteonectin [12] and BM-40 [13], is a matricellular protein that is expressed intracellularly and is secreted into the extracellular matrix (ECM). It functions, in part, to regulate levels of cell adhesion and cell migration, as well as to regulate cell proliferation, survival, and angiogenesis [14-16]. These functions are important for normal development and for physiological processes such as tissue remodeling during wound healing [14,15]. Its function is mediated, in part, through the manipulation of integrin-ECM interactions [17,18], which in turn can influence growth factor-induced signaling cascades. Its function, therefore, is influenced by the integrin expression profile of the cells, the ECM present in the microenvironment, and the growth factor-growth factor receptor status. As a consequence, its role might differ between tissues or even from location to location within a tissue, depending on the microenvironment. This is important to consider because the role of SPARC in cancer is somewhat controversial, as it positively correlates with invasion or worse prognosis for some cancers, but negatively correlates with invasion or worse prognosis for others [19]. As a result, it has been regarded as a therapeutic target for pancreatic adenocarcinoma [20] and gastric cancer [21] on the one hand, but as a therapeutic agent for colorectal [22,23] and ovarian [24] cancers on the other. Indeed, in ovarian cancer, SPARC has been shown to sensitize tumor cells to cisplatin therapy [24] and to enhance apoptosis and potentiate sensitivity to the chemotherapeutic agent 5-fluorouracil in colorectal cancer [23]. In the latter, this sensitivity was mediated by SPARC binding to procaspase 8.

We previously demonstrated that SPARC protein is not immunohistochemically detectable in normal human cerebral cortex but is highly expressed in human astrocytomas grades II-IV [25]. A subsequent study showed SPARC to have restricted expression to the marginal glia of the outer layer of the cortex, Bergmann glia in the cerebellum, and an unidentified subpopulation of cells in the subcortical white matter, and to be highly expressed in all grades of astrocytomas [26].

We further demonstrated that SPARC promotes tumor cell migration and invasion *in vitro *[27,28], and we and others have demonstrated that SPARC promotes invasion *in vivo *[29,30], suggesting that it is a therapeutic target to prevent tumor invasion of gliomas. In addition, we have shown that SPARC expression decreases glioma proliferation [29], and in this respect SPARC expression is advantageous. Therefore, using SPARC as a therapeutic target could result in the desired decrease of tumor invasion, but might also result in an undesired increase in tumor proliferation. We have therefore investigated the signaling pathways induced by SPARC to identify potential downstream therapeutic targets to specifically inhibit SPARC-induced invasion, while maintaining SPARC-mediated inhibition of proliferation.

We have found that SPARC promotes glioma migration and invasion, in part, through the upregulation of the p38 MAPK-MAPKAPK2 (MK2)-HSP27 signaling axis [28]. The small heat shock protein 27 (HSP27) contributes to actin microfilament stabilization and reorganization needed for cell migration [31,32]. These functions are dependent upon its phosphorylation status [31,32]. Indeed, we demonstrated that treatment of SPARC-expressing glioma cells with HSP27 siRNA prevented SPARC-induced migration and invasion [28].

Interestingly, SPARC also promotes glioma cell survival under stressful conditions by upregulating AKT activity [33]. The activation of AKT is thought to be through the binding of SPARC to integrin beta 1 subunit [17,18,34], and downstream activation of ILK [34]. Activated ILK activates AKT [35]. Indeed, suppression of SPARC is accompanied by decreased ILK activity [36].

In addition, HSP27 and AKT exist in complex with p38 MAPK and MAPKAPK2 in the cytoplasm [37]. Activation of p38 MAPK results in the downstream activation of MAPKAPK2, which phosphorylates HSP27 [37]. pHSP27 can bind to AKT and act as a scaffold protein to permit the phosphorylation of AKT by MAPKAPK2 [38], leading to enhanced tumor cell survival signaling by mTOR activation and downstream suppression of autophagy [39]. As SPARC can potentially promote AKT survival signaling through ILK and/or HSP27, we hypothesized that HSP27 may serve as a downstream target, not only to inhibit SPARC-induced migration and invasion, but also to eliminate SPARC-induced tumor cell survival signaling through AKT activation.

HSP27 also plays a major role in inhibiting extrinsic and intrinsic cell death pathways. It inhibits the extrinsic apoptotic signaling pathway by preventing DAAX-mediated signaling [40], and can prevent extrinsic and intrinsic pathways by inhibiting the translocation of pro-apoptotic tBID onto the mitochondrial membrane [40]. In addition, it can inhibit intrinsic apoptotic signaling by binding to cytosolic cytochrome C and thereby prevent the formation of the apoptosome and caspase 9 activation [41-45]. By interfering with caspase 3 activation, it indirectly also limits caspase 7 activation [45]. Therefore, the inhibition of HSP27 is expected to promote apoptotic signaling, as well as inhibit SPARC-induced tumor cell survival signaling.

Consequently, the goals of this study were to determine 1) whether SPARC sensitized glioma cells to radiation or chemotherapy, 2) whether targeting SPARC decreased tumor cell survival, 3) whether HSP27 inhibition was a better target to suppress SPARC-induced glioma cell survival, and 4) determine whether HSP27 inhibition suppressed SPARC-induced AKT activation and survival. To this end, 1) control- and SPARC-expressing U87 cells or LN443 cells treated with control or SPARC siRNAs were untreated or subjected to TMZ or radiation therapy (RT), and 2) control- and SPARC-expressing U87 cells, LN443 cells, and human primary glioma cell lines treated with control, HSP27, SPARC, or AKT siRNAs or AKT inhibitor IV were subjected to Western blot analyses to assess tumor cell survival and death signaling, and were subjected to clonogenic assays to determine whether the treatments affect tumor cell survival and/or sensitize tumor cells to TMZ treatment.

## Results

### SPARC expression has no effect on glioma colony forming efficiency or response to RT

The current treatment regimen for glioma patients includes RT. If SPARC status affects RT outcome, this would be important to know when considering targeting SPARC or its downstream signaling molecules for therapy. Therefore, clonogenic assays were used to evaluate the effects of SPARC on RT using our previously described U87 cells (low endogenous SPARC expression) transfected with control GFP or SPARC-GFP fusion protein [28] or LN443 cells (high endogenous SPARC expression) transfected with control or SPARC siRNA.

**Figure 1:**
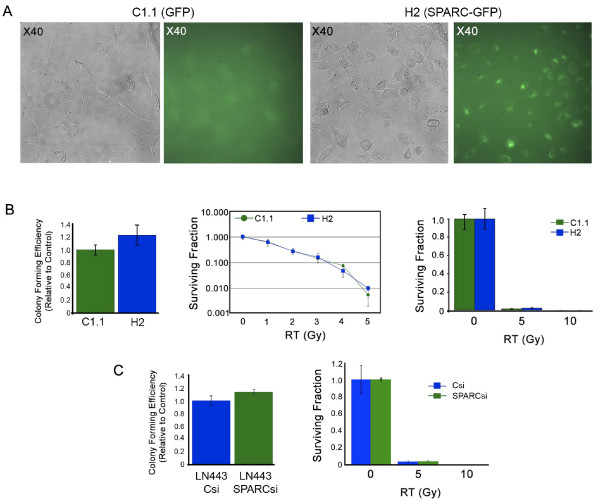
**SPARC does not enhance or suppress radiation therapy (RT)**. **A**. Representative fluorescent images of control C1.1 GFP- and H2 SPARC-GFP-expressing clonogenic colonies (×40 magnification). **B**. Average colony forming efficiency ± SD of untreated C1.1 and H2 cells (left panel). Average surviving fraction ± SD of C1.1 and H2 cells exposed to 0 or 1-5 Gy (middle panel) or 0, 5 or 10 Gy (right panel), plating 3000 cells/60-mm dish. Plating 1000 cells/60-mm dish gave similar results. **C**. Average colony forming efficiency ± SD of untreated LN443 cells transfected with control (Csi) or SPARC siRNA (SPARCsi) (left panel). Average surviving fraction ± SD of LN443 cells transfected with Csi or SPARCsi exposed to 0, 5 or 10 Gy (right panel) plating 750 cells/60-mm dish. Plating 1500 cells/60-mm dish gave similar results.

Fluorescence imaging showed that the surviving U87-transfected colonies expressed either GFP (C1.1) or SPARC-GFP (H2; Figure 1A). The clonogenic assay indicated that enhancing SPARC expression in these cells did not alter colony forming efficiency or alter survival in response to RT (Figure 1B). In a complementary experiment, suppressing SPARC expression in LN443 cells using SPARC siRNA also had no effect on the colony forming efficiency or survival response to RT of these cells (Figure 1C). Therefore, SPARC status does not alter the effects of RT, suggesting it cannot be used as a therapy to enhance radiation sensitivity.

### Forced SPARC expression protects tumor cells against TMZ

**Figure 2:**
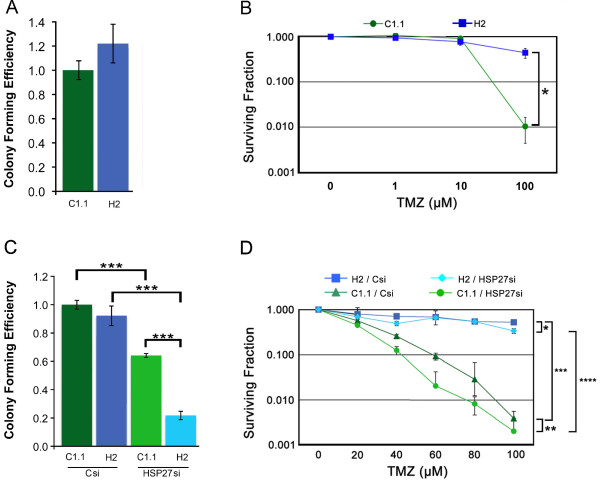
**Forced SPARC expression protects against temozolomide (TMZ) and suppresses the ability of HSP27 inhibition to sensitize cells to lower concentrations of TMZ**. **A**. Average colony forming efficiency ± SD of C1.1 and H2 plating 750 cells/60-mm dish. **B**. Average surviving fraction ± SD of C1.1 GFP- and H2 SPARC-GFP-expressing cells in 0 (0.1% DMSO), 1, 10, or 100 μM TMZ plating 750 cells/60-mm dish. TMZ (100 μM) suppressed survival of both clones relative to 0 drug; p ≤ 0.0048. However, SPARC-expressing cells survived better than control cells in 100 μM TMZ; * p = 0.0022. Plating 375 and 1500 cells gave similar results. **C**. Average colony forming efficiency ± SD of C1.1 and H2 cells transfected with control (Csi) or HSP27 siRNA (HSP27si), plating 750 cells/60-mm dish. *** p = 0.0001. **D**. Average surviving fraction ± SD of C1.1 and H2 cells transfected with Csi or HSP27si in the absence (0) or presence of increasing concentrations of TMZ, plating 750 cells/60-mm dish. * H2 + Csi vs. H2 + HSP27si: 100 μM TMZ; p = 0.0020. ** C1.1 + Csi vs. C1.1 + HSP27si: 20 μM TMZ; p = 0.022, 40 μM TMZ; p = 0.0026, 60 μM and 80 μM TMZ; p = 0.0001. *** H2 + Csi vs. C1.1 + Csi: 20 μM TMZ; p = 0.015, 40-100 μM TMZ; p = 0.0001. **** H2 + HSP27si vs. C1.1 + HSP27si: 20-60 μM TMZ; p ≤ 0.0055, 80 μM and 100 μM TMZ; p = 0.0002. B, C. Plating 1500 cells gave similar results.

We then determined whether SPARC alters the surviving fraction of glioma cells treated with TMZ (Figure 2). C1.1 GFP- and H2 SPARC-GFP-expressing glioma cells were treated with increasing concentrations of TMZ for 2 days. Media were changed and the ability of cells to form colonies was assessed by clonogenic assay. In agreement with data in Figure 1, the colony forming efficiencies of untreated control and SPARC-expressing cells were similar (Figure 2A). For C1.1 control cells, 100 μM TMZ treatment severely reduced the surviving fraction (100-fold). In contrast, the H2 SPARC-expressing cells survived better, with 100 μM TMZ decreasing the surviving fraction only 2.3-fold. Importantly, these data indicate that SPARC-expressing tumor cells survive better in TMZ (44-fold).

### HSP27 inhibition suppresses survival more effectively in SPARC-expressing cells

To determine whether targeting HSP27 had differential effects in the absence or presence of SPARC, C1.1 GFP- and H2 SPARC-GFP-expressing glioma cells were treated with control or HSP27 siRNAs. The colony forming efficiencies of both cell lines treated with control siRNA were similar (Figure 2C). HSP27 siRNA suppressed the colony forming efficiency of control cells (1.6-fold). However, treatment with HSP27 siRNA suppressed the colony forming efficiency of SPARC-expressing cells even more (4.2-fold; Figure 2C). These data suggest that inhibition of HSP27 decreases tumor cell survival, and inhibition is even more effective in SPARC-expressing cells.

### Combined HSP27 inhibition and TMZ treatment is less effective in SPARC-expressing cells

To determine whether HSP27 mediates SPARC-induced survival in TMZ, C1.1 GFP- and H2 SPARC-GFP-expressing glioma cells were treated with control or HSP27 siRNAs followed by treatment with increasing concentrations of TMZ for 2 days. Media were changed and the ability of cells to form colonies was assessed by clonogenic assay (Figure 2D).

TMZ alone suppressed survival of the control siRNA-treated SPARC-expressing cells approximately 2-fold (p = 0.0004), while HSP27 inhibition plus 100 μM TMZ only modestly suppressed the surviving fraction of the SPARC-expressing cells a further 1.6-fold. TMZ alone suppressed survival of the control siRNA-treated GFP-expressing cells approximately 120-fold, while the inhibition of HSP27 plus TMZ increased the sensitivity of the control cells to lower doses (40-80 μM) of TMZ (Figure 2D). These data indicate that HSP27 inhibition sensitizes glioma cells to TMZ, but more so in the absence of SPARC. Therefore despite the fact that SPARC-expressing cells were more susceptible to HSP27 inhibition alone, combined HSP27 siRNA and TMZ was not as effective in these cells.

### SPARC enhances the expression or activation of pro-survival and pro-death proteins

**Figure 3:**
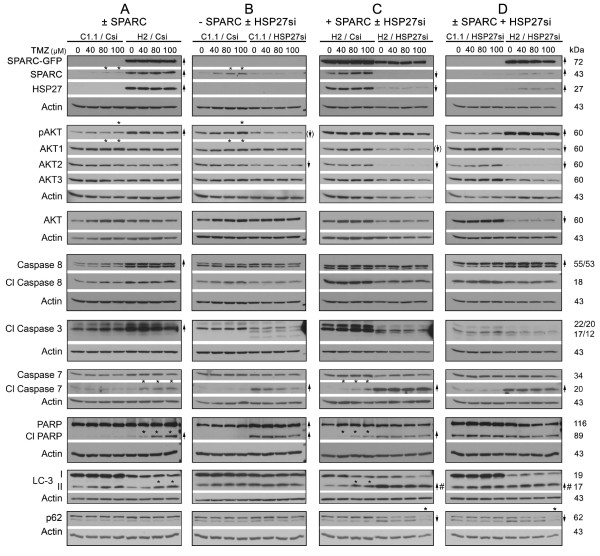
**Forced SPARC enhances the expression or activation of pro-survival and pro-death proteins but promotes death signaling in temozolomide (TMZ), whereas HSP27 inhibition promotes apoptotic signaling (SPARC-independent) and autophagic signaling (SPARC-dependent), but suppresses autophagic signaling in the presence of TMZ and SPARC**. **A-D**. Representative Western blots are illustrated for n ≥ 3 experiments of C1.1 control GFP- or H2 SPARC-GFP-expressing cells treated with control siRNA (Csi) or HSP27 siRNA (HSP27si) for 72 hr. Equal numbers of siRNA-treated cells were plated overnight in growth serum, and then treated with 0 (0.1% DMSO control), 40, 80 or 100 μM TMZ for 2 days before lysing. Arrows indicate ≥ 2-fold increases or decreases due to ± SPARC expression and/or ± HSP27 siRNA treatment. Arrow with brackets indicates 30% reduction. # - Indicates a ≥ 2-fold increase in the ratio of LC-3II/LC-3I. Asterisks indicate ≥ 2-fold increases or decreases due to TMZ treatment.

To better understand the mechanism by which SPARC promotes survival and protects cells against TMZ and how HSP27 inhibition suppresses survival in the absence or presence of SPARC and/or TMZ, Western blots of lysates from C1.1 GFP- and H2 SPARC-GFP-expressing cells treated with control or HSP27 siRNAs were probed for survival and death associated proteins. As TMZ has been implicated in both apoptotic [46] and autophagic [47] death in glioma cells, both mechanisms were investigated (Figure 3). An initial timing study was performed to determine the effects of TMZ on control cells (see Additional file 1: Figure S1). By days 6 and 8, no increase in PARP cleavage was observed; however, TMZ did induce autophagy, as detected by an increase in LC3-II and increased p62 degradation, inferred by a concomitant decrease in p-p62 (upper band) and increased unphosphorylated p62 (lower band). These data suggest that TMZ-induced autophagy is the major death mechanism in these cells, at least at the time points examined, and is likely responsible for the approximate 120-fold decrease in the surviving fraction observed for the Csi-treated C1.1 control cells treated for 2 days with 100 μM TMZ (Figure 2D).

To determine whether SPARC alters survival and death signaling, Westerns of lysates from control siRNA-treated C1.1 GFP-expressing and the H2 SPARC-GFP-expressing cells were compared (0 vs. 0 TMZ; Figure 3A). The data show, as previously reported, that SPARC-GFP promotes the upregulation of endogenous SPARC [28], HSP27 [28], and pAKT [33]. This increase in pro-survival proteins was accompanied by increased procaspase 8 and a less than 2-fold increase in cleaved caspase 8, and by enhanced cleavage of caspase 3 to p22 and p20 fragments. These changes were accompanied by a very slight signal for cleaved PARP (Figure 3A). SPARC had no effect on autophagy based on LC3-II and p62 levels. Therefore, SPARC regulates both pro-survival and pro-apoptotic proteins, but their increases in expression appear to counterbalance one another as the C1.1 control GFP- and H2 SPARC-GFP-expressing cells treated with control siRNA have similar colony forming efficiencies (Figure 2C and see Additional file 2: Figure S2, Panel A for proposed signaling mechanisms).

### SPARC promotes apoptotic signaling in the presence of TMZ

Interestingly, two days of TMZ treatment (80-100 μM) slightly increased endogenous SPARC, pAKT, and AKT1 levels in C1.1 control cells (Figure 3A and 3B); however these effects were not observed in SPARC-GFP-transfected cells (Figure 3A and 3C). Rather, SPARC expression combined with increasing concentration of TMZ resulted in increasing caspase 7 and PARP cleavage (Figure 3A, Figure 3C and see Additional file 2: Figure S2, Panel A for proposed signaling mechanisms). This increase in apoptotic signaling likely contributes to the 2-fold decrease in the surviving fraction of the control siRNA (Csi)-treated SPARC-expressing cells with 100 μM TMZ (Figure 2D).

The slight increase in LC3-II in the H2 SPARC-GFP-expressing cells (2.5-fold) compared to that in the GFP-expressing cells (1.7-fold) likely does not contribute, as no changes in expression were observed for p62. These data suggest that the increases in LC3-II represent initiation of TMZ-induced autophagy at this time point, and that SPARC does not enhance autophagy.

Therefore, although SPARC expression enhanced pro-apoptotic signaling after 2 days in TMZ, the Western results combined with the clonogenic survival data suggest that the SPARC-induced upregulation of the pro-survival HSP27 and/or pAKT proteins may counter the upregulation of the pro-death signals, thereby permitting better survival in TMZ compared to the control cells.

### HSP27 inhibition enhances apoptotic signaling independently of forced SPARC, and enhances autophagic signaling in the presence of forced SPARC

To determine whether the inhibition of HSP27 could shift the balance of SPARC-induced signaling towards increased death signaling, C1.1 control cells (Figure 3B) and H2 SPARC-expressing cells (Figure 3C) were treated with control or HSP27 siRNAs.

As expected, no HSP27 signal was observed in control cells treated with either control or HSP27 siRNAs due to the very low level of endogenous HSP27 (Figure 3B; 0 vs. 0 TMZ). Despite this, HSP27 siRNA treatment was accompanied by decreased AKT2, and a 30% decrease in pAKT, suggesting that in control cells, the low level of endogenous HSP27 (observed in long film exposures) regulated pAKT activation. The loss of HSP27 did not affect caspase 8, but was accompanied by caspase 3 cleavage to active p17 and p12 fragments, and increased cleavage of caspase 7 and PARP. No changes were observed in LC-3II/LC-3I ratio. Therefore, in the absence of forced SPARC, HSP27 inhibition suppresses survival signaling and induces apoptotic signaling.

In the H2 SPARC-expressing cells, HSP27 siRNA treatment significantly reduced HSP27 as expected (Figure 3C; 0 vs. 0 TMZ). Of note, inhibition of HSP27 was accompanied with suppressed levels of endogenous SPARC and a decrease in AKT1 and 2 by 30% and 80%, respectively. Despite a decrease in total AKT, pAKT level was unchanged, suggesting that forced SPARC maintained AKT phosphorylation. Similar to control cells, the loss of HSP27 did not affect caspase 8, and was accompanied by caspase 3 cleavage to active p17 and p12 fragments, and increased cleavage of both caspase 7 and PARP. In contrast to the control cells, HSP27 inhibition was accompanied by an increase in LC-3II and a higher LC-3II/LC-3I ratio in the SPARC-expressing cells. The induction of autophagy was also supported by a decrease in p-p62 (upper band), suggesting degradation of p-p62, and an increase in p62 (lower band), suggesting synthesis of p62 to maintain autophagy.

To assess the effects of HSP27 inhibition in the absence versus the presence of forced SPARC expression, a direct comparison of control and SPARC-expressing cells treated with HSP27 siRNA is illustrated (Figure 3D). This comparison confirmed that SPARC maintains elevated pAKT despite the greater than 2-fold decreases in AKT1 and 2, that HSP27 inhibition induces apoptosis independently of SPARC, and that autophagy is enhanced in the presence of SPARC. These data suggest that the further decrease in colony forming efficiency in SPARC-expressing cells versus control cells treated with HSP27 siRNA (Figure 2C) is due to autophagy.

### HSP27 inhibition combined with TMZ suppresses autophagy in SPARC-expressing cells

The changes in apoptotic signaling induced by HSP27 siRNA were not altered by TMZ in either the control or SPARC-expressing cells (Figure 3B, C, D and see Additional file 2: Figure S2, Panel B for proposed signaling mechanisms). However, HSP27 inhibition combined with TMZ treatment appears to suppress autophagy in SPARC-expressing cells as evidenced by a decrease in both p62 (lower band) and p-p62 (upper band; Figure 3C, D). These results suggest that the maintenance of high pAKT by forced SPARC expression promotes the survival in TMZ observed by the clonogenic assay (Figure 2D). We therefore determined whether inhibition of AKT phosphorylation could sensitize the forced SPARC-expressing cells to TMZ.

### Suppression of pAKT signaling induces autophagic signaling in control and SPARC-expressing cells in TMZ

**Figure 4:**
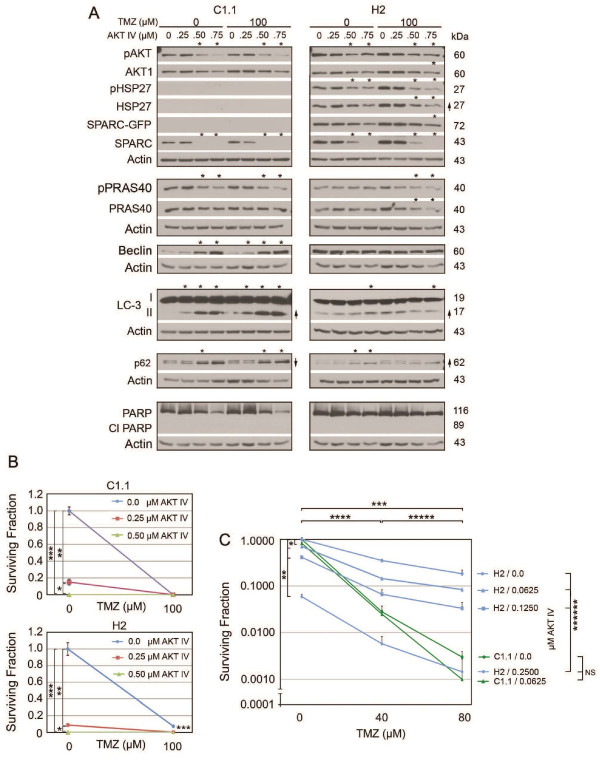
**Inhibition of pAKT eliminates SPARC-induced survival in temozolomide (TMZ)**. **A**. Representative Western blots of C1.1 control GFP- or H2 SPARC-GFP-expressing cells in the absence 0 (0.1% DMSO control) or presence of 100 μM TMZ ± AKT inhibitor IV at indicated concentrations for 48 hr before lysing. Arrows indicate ≥ 2-fold increases or decreases due to ± TMZ treatment and asterisks indicate ≥ 2-fold increases or decreases due to AKT inhibitor IV treatment. **B**. Means for surviving fraction ± SD of C1.1 or H2 cells treated ± AKT IV inhibitor ± TMZ plating 1500 cells/60-mm dish are presented. For C1.1 * p = 0.0022, **, *** p = 0.0001. For H2 *,**,*** p = 0.0001. **C**. Means for surviving fraction ± SD of C1.1 or H2 cells treated ± AKT IV inhibitor ± TMZ plating 1500 cells/60-mm dish are presented. * p = 0.014, ** p = 0.0006, *** p ≤ 0.0001, **** p ≤ 0.0003, ***** p ≤ 0.03 except H2 0.25 AKT inhibitor IV (40 vs. 80 ≤ μM TMZ) is not significant, ****** p ≤ 0.008. NS - not significant. Plating 3000 cells gave similar results.

AKT inhibitor IV was used to inhibit pAKT signaling in C1.1 GFP- and H2 SPARC-GFP expressing cells in the absence and presence of 100 μM TMZ (Figure 4). The decrease in pPRAS40 confirmed the inhibition of pAKT in control cells (Figure 4A). This pAKT suppression was accompanied by increased autophagic signaling as assessed by increased beclin and LC3-II, and a concomitant decrease in p-p62 (upper band) and increase in p62 (lower band; Figure 4A). Inhibition of pAKT did not induce apoptosis. TMZ alone was less effective at inducing autophagic signaling, and had no effect on pAKT inhibition.

In H2 SPARC-expressing cells, pAKT IV inhibition decreased the level of pAKT, but was not as efficient at inhibiting downstream signaling as the pPRAS40 levels remained unchanged. As a result, 0.50 μM and 0.75 μM AKT IV induced autophagy, but to a lesser degree. This is likely due to forced SPARC expression maintaining a higher level of pAKT in these cells. Despite this, the inhibitor-induced autophagic signaling was still greater than that observed in cells treated with TMZ alone, suggesting that the inhibitor should eliminate SPARC-induced survival in TMZ.

### AKT IV inhibitor suppresses colony forming efficiency and eliminates SPARC-induced survival in TMZ

In corresponding clonogenic assays, 0.5 μM AKT inhibitor IV was able to suppress the colony forming efficiency of both control and SPARC-expressing cells (Figure 4B). The AKT IV inhibitor was as effective as 100 μM TMZ alone for control cells (Figure 4B, top panel). Furthermore, the same concentration of inhibitor eliminated the survival advantage of SPARC-expressing cells in TMZ (Figure 4B, Bottom panel). To more accurately assess the response of cells to TMZ after pAKT inhibition, lower doses of AKT inhibitor IV were used with lower doses of TMZ. AKT inhibitor IV did further sensitize the cells to TMZ, and 0.25 μM AKT inhibitor IV in combination with 80 μM TMZ was able to suppress the survival of SPARC-expressing tumor cells to that observed for control cells treated with TMZ alone (Figure 4C). These data suggest that SPARC-induced upregulation of pAKT does lead to better survival in TMZ.

The combined data thus far indicate that SPARC promotes both pro-survival and pro-apoptotic signaling that favors maintained survival. Inhibiting HSP27 is effective in both control and SPARC-expressing cells by inducing apoptosis in control cells and apoptosis and autophagy in SPARC-expressing cells. Although SPARC induces apoptotic signaling in TMZ, its induced pro-survival signaling predominates to protect cells against temozolomide. This protection can be removed by suppressing SPARC-induced upregulation of pAKT activity.

It is interesting to note that forced SPARC expression increases HSP27 and pAKT (Figure 3A), but the inhibition of HSP27 suppressed SPARC and pAKT in the C1.1 control cells (Figure 3B), and endogenous SPARC in the H2 cells (Figure 3C). This suggests that HSP27 and SPARC have the potential to regulate each other, but this regulation is disrupted in the presence of forced SPARC. To determine whether HSP27 regulates SPARC and pAKT in the absence of forced SPARC, we examined the effects of HSP27 inhibition on LN443 cells, which have high endogenous SPARC expression.

### HSP27 inhibition in LN443 cells suppresses SPARC and pAKT expression, promotes death signaling, decreases colony forming efficiency, and increases sensitivity to TMZ

**Figure 5:**
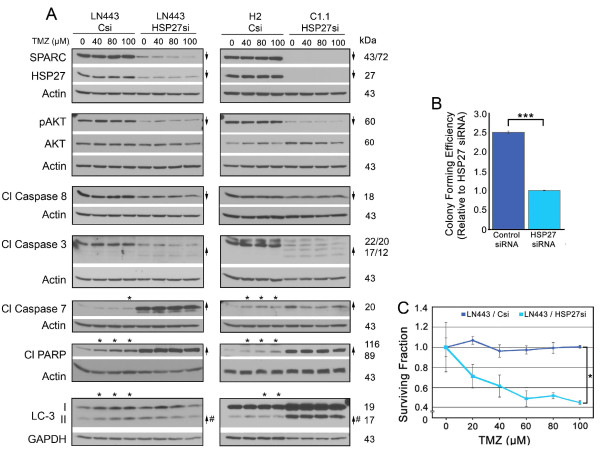
**Inhibition of HSP27 in LN443 cells suppresses survival signaling, promotes death signaling, and decreases colony forming efficiency, eliminates SPARC-induced pro-death signaling in temozolomide (TMZ), and decreases survival in TMZ**. **A**. Representative Western blots of LN443 cells (left panel) treated with control siRNA (Csi) or HSP27 siRNA (HSP27si) and H2 cells treated with control siRNA (Csi) and C1.1 cells treated with HSP27 siRNA (HSP27si) (right panel) for 72 hr. For both panels, equal numbers of siRNA-treated cells were plated overnight in growth serum, and then treated with 0 (0.1% DMSO control), 40, 80 or 100 μM TMZ for 2 days before lysing. Arrows indicate ≥ 2-fold increases or decreases due to HSP27 siRNA treatment. # - Indicates a ≥ 2-fold increase in the ratio of LC-3II/LC-3I. Asterisks indicate ≥ 2-fold increases or decreases due to TMZ treatment. Note: SPARC refers to endogenous SPARC in LN443 cells and SPARC-GFP in H2 cells, and 43/72 refers to the 43 kDa endogenous SPARC, whereas 72 kDa refers to SPARC-GFP. **B**. Means for colony forming efficiency ± SD of LN443 cells ± HSP27 siRNA plating 750 cells/60-mm dish. *** p = 0.018. **C**. Means for surviving fraction ± SD of LN443 cells ± HSP27 siRNA ± increasing concentrations of TMZ plating 750 cells/60-mm dish. * Csi vs. HSP27si: 20 μM; p = 0.0031, 40 μM; p = 0.0092, 60 μM; p = 0.0006, 80 μM; p = 0.0011, and 100 μM; p = 0.0053. Plating 1500 cells gave similar results.

LN443 glioma cells are highly resistant to TMZ treatment (Additional file 3: Figure S3), and have high SPARC, HSP27 and pAKT expression (Figure 5, Panel A). We proposed that the inhibition of HSP27, in the absence of forced SPARC, should suppress SPARC and pAKT expression and induce death signaling. Further we proposed that the presence of SPARC in LN443 control siRNA-treated cells should correlate with TMZ-induced death signaling that would be eliminated by HSP27 inhibition. Finally, we proposed that HSP27 inhibition should decrease colony forming efficiency and increase sensitivity to TMZ.

LN443 cells were treated with control and HSP27 siRNAs. As predicted, HSP27 siRNA treatment did suppress SPARC and pAKT levels, as well as decrease caspase 8 cleavage (Figure 5A; left panel, 0 vs. 0 TMZ). In addition, inhibition of HSP27 increased caspase 3, caspase 7 and PARP cleavage. As the LC-3II/LC-3I was slightly increased, likely due to suppression of pAKT, the data suggest that both autophagy and apoptosis may be induced by HSP27 inhibition in these cells. Indeed, the colony forming efficiency was suppressed approximately 2.5 fold by HSP27 siRNA treatment (Figure 5B).

In agreement with the C1.1 and H2 data, high SPARC expression in control siRNA-treated LN443 cells correlated with increased caspase 7 and PARP cleavage, and increased LC3-II in the presence of TMZ (Figure 5A; left panel). Furthermore, this sensitivity to TMZ-induced death signaling by SPARC was eliminated by treatment with HSP27 siRNA (Figure 5A, left panel and see Additional file 2: Figure S2, Panel C for proposed signaling mechanisms). The suppression of pAKT in LN443, as a result of blocking HSP27, correlated with a 2-fold increase in sensitivity to TMZ (Figure 5C).

Based on these data, we reasoned that the expression profiles of control siRNA-treated LN443 cells (high HSP27, high SPARC) versus the HSP27 siRNA-treated LN443 cells (low HSP27, low SPARC) should be equivalent to the expression profiles observed for control siRNA-treated H2 cells (high HSP27, high SPARC) versus HSP27 siRNA-treated C1.1 cells (low HSP27, low SPARC). Indeed, the results were similar (Figure 5A, right panel), indicating that the results are not cell-line specific.

Therefore, HSP27 inhibition is also efficient in inducing death signaling in these glioma cells, and similar to C1.1 cells (low SPARC, low pAKT) inhibition increased sensitivity to lower doses of TMZ. Unfortunately, this experiment could not determine whether the decrease in pAKT was directly due to inhibition of HSP27 or consequential to HSP27 siRNA-induced suppression of SPARC. Therefore, we next determined whether targeting SPARC would also produce the same results.

### Inhibition of SPARC decreases apoptotic signaling and eliminates sensitivity to TMZ in LN443 cells, but enhances colony forming efficiency

To determine whether inhibition of SPARC would mimic inhibition of HSP27, LN443 cells were similarly subjected to control and SPARC siRNAs and the effects on downstream signaling, colony forming efficiency, and tumor cell survival in TMZ were similarly evaluated.

**Figure 6:**
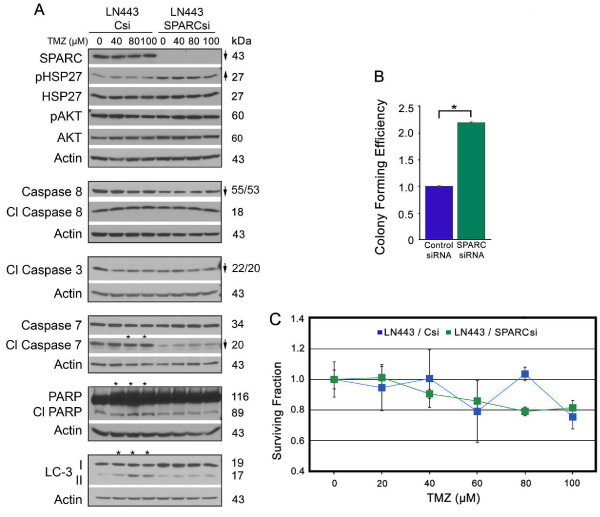
**Inhibition of SPARC expression in LN443 suppresses pro-apoptotic signaling, increases colony forming efficiency, eliminates SPARC-induced pro-death signaling in temozolomide (TMZ), and has no effect on survival in TMZ**. **A**. Representative Western blots of LN443 cells treated with control siRNA (Csi) or SPARC siRNA (SPARCsi) for 72 hr. Equal numbers of siRNA-treated cells were plated overnight in growth serum, and then treated with 0 (0.1% DMSO control), or 40, 80 or 100 μM TMZ for 2 days before lysing. Arrows indicate ≥ 2-fold increases or decreases due to SPARC siRNA treatment. Asterisks indicate ≥ 2-fold increases or decreases due to TMZ treatment. **B**. Means for colony forming efficiency ± SD of LN443 ± SPARC siRNA plating 375 cells/60-mm dish. * p = 0.018. **C**. Means for surviving fraction ± SD of LN443 ± SPARC siRNA ± increasing concentrations of TMZ plating 375 cells/60-mm dish. Plating 750 cells gave similar results.

As expected, the loss of SPARC decreased procaspase 8, cleaved caspase 3 p22/20, and cleaved caspase 7, which was accompanied by a lack of PARP cleavage (Figure 6A). The inhibition of SPARC had no effect on total HSP27, AKT, and pAKT, and was accompanied by increased levels of pHSP27, supporting the contention that SPARC is downstream of HSP27 signaling in these cells, and that HSP27 and AKT induce survival (see Additional file 2: Figure S2, Panel D for proposed signaling mechanisms). The loss of SPARC and its induced apoptotic signaling combined with the maintenance of HSP27 and AKT pro-survival signaling shifted the balance to increase survival as assessed by colony forming efficiency (Figure 6B).

As previously demonstrated, SPARC expression was associated with death signaling in TMZ, and SPARC siRNA treatment suppressed this signaling (Figure 6A), demonstrating that SPARC is indeed required for this response. In agreement with the previous data, this enhanced signaling in TMZ had little effect on cell survival in TMZ (Figure 6C).

That inhibition of SPARC had no effect on HSP27 or pAKT in these cells supports the suggestion that HSP27 regulates SPARC and pAKT independently in these cells. When SPARC is inhibited, HSP27 and pAKT inhibit apoptosis and autophagy, and SPARC-induced death signaling in TMZ is eliminated, resulting in greater survival of cells. These data indicate that SPARC is not a good therapeutic target in these cells, and reinforces the conclusion that SPARC is a poor chemosensitizer in TMZ.

### Suppression of total AKT1/2 enhances colony forming efficiency and suppression of AKT1/2 or AKT3 reduces SPARC-induced death signaling in TMZ

HSP27 inhibition suppressed pAKT in the absence of SPARC in both the control C1.1 cells (Figure 3) and in the LN443 cells (Figure 5), and this correlated with increased apoptotic signaling. In addition, inhibition of pAKT using inhibitor IV increased autophagy and decreased clonogenic survival (Figure 4). These data suggest that HSP27-induced activation of AKT contributes to survival. However, inhibition of HSP27 affected AKT1 and 2 differently than AKT3 depending on SPARC status (Figure 3). We therefore separately examined the effects of control, AKT1/2 or AKT3 siRNAs on downstream signaling, colony forming efficiency, and survival in TMZ in the LN443 cells.

**Figure 7:**
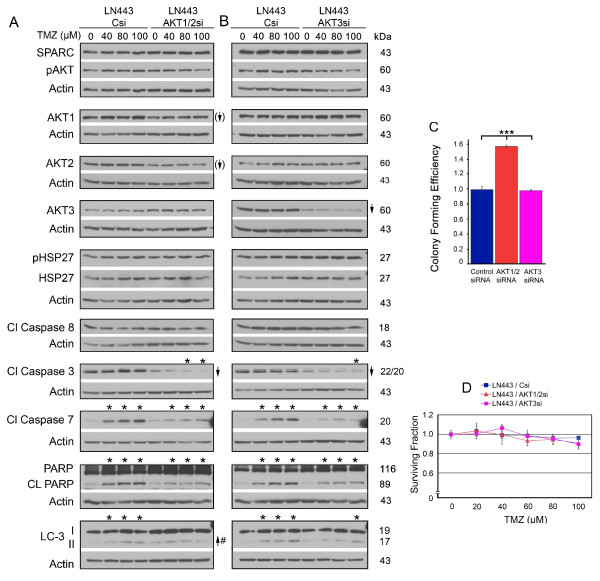
**Suppression of AKT1/2 or AKT3 decreases SPARC-induced pro-death signaling in temozolomide (TMZ); suppression of AKT1/2 increases colony forming efficiency, and has no effect on survival in TMZ**. **A, B**. Representative Western blots of LN443 cells treated with control siRNA (Csi), AKT1/2 siRNA (AKT1/2si) or AKT 3 siRNA (AKT3si) for 72 hr. Equal numbers of siRNA-treated cells were plated overnight in growth serum, and then treated with 0 (0.1% DMSO control), or 40, 80 or 100 μM TMZ (TMZ) for 2 days before lysing. Arrows indicate ≥ 2-fold increases or decreases due to HSP27 siRNA treatment. Arrows with brackets indicate 30% and 40% reduction of AKT1 and AKT2, respectively. # - Indicates a ≥ 2-fold increase in the ratio of LC-3II/LC-3I. Asterisks indicate ≥ 2-fold increases or decreases due to TMZ treatment. **C**. Means for colony forming efficiency ± SD for LN443 cells ± AKT1/2 or AKT3 siRNA plating 750 cells. *** p ≤ 0.0003. **D**. Means for surviving fraction ± SD for LN443 cells ± AKT1/2 or AKT3 siRNA ± increasing concentrations of TMZ plating 750 cells/60-mm dish.

It was difficult to suppress total AKT1 and AKT2 more than 30% and 40%, respectively. This level of inhibition did not suppress pAKT levels (Figure 7A), and so the data were used to determine effects of reducing the total AKT levels. This level of suppression did not affect HSP27 or SPARC levels, suggesting the total AKT inhibition effects are downstream of SPARC and HSP27. Interestingly, the suppression of AKT1/2 had little effect on autophagic signaling or PARP cleavage despite the unanticipated decrease in caspase 3 cleavage (0 TMZ Csi to 0 TMZ AKT1/2si, Figure 7A). This lack of death signaling was accompanied by increased colony forming efficiency (Figure 7C). This result was similar to that observed when inhibiting SPARC, suggesting that AKT1/2 may mediate this aspect of SPARC regulation of survival. That SPARC and AKT1/2 may function together in some as yet unknown mechanism is reinforced by the observation that suppression of AKT1/2 also reduced SPARC-induced death signaling in TMZ (Figure 7A).

Similar signaling results were observed using AKT3 siRNA, which was very efficient at reducing AKT3 levels (Figure 7B). While no changes were observed in colony forming efficiency due to inhibition of AKT3 (Figure 7C), suppression of SPARC-induced death signaling in TMZ was also observed (Figure 7B and see Additional file 2: Figure S2, Panel E for proposed signaling mechanisms). These data suggest that the AKTs contribute to SPARC-induced sensitivity to TMZ, and confirms that this signaling has little effect, as assessed by the clonogenic assay (Figure 7D).

### Inhibition of pAKT decreases SPARC and increases autophagy

**Figure 8:**
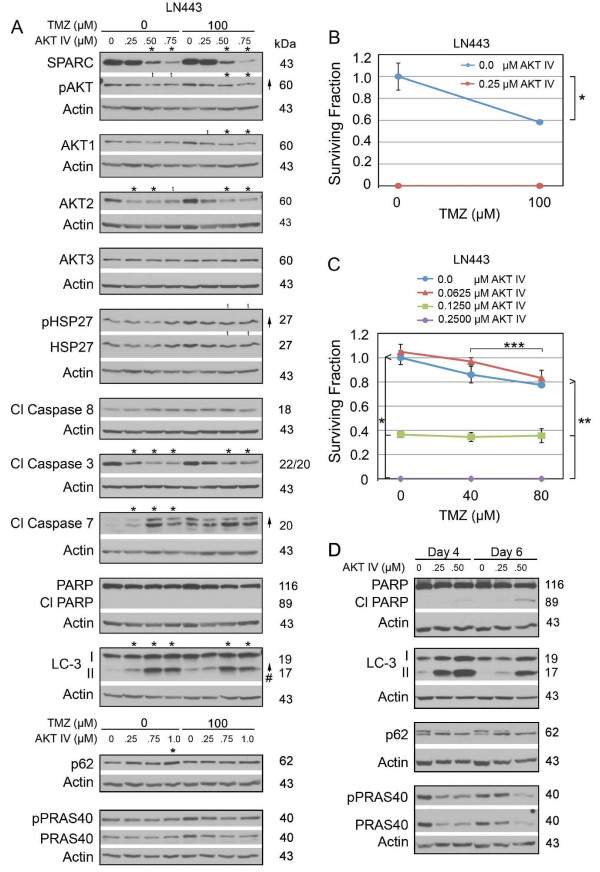
**Suppression of pAKT in LN443 enhances autophagic signaling, and decreases survival of LN443 cells**. **A**. Representative Western of LN443 cells treated with 0 (0.1% DMSO) or 100 μM TMZ ± AKT inhibitor IV at the concentrations indicated for 2 days before lysing. Arrows indicate ≥ 2-fold increases or decreases due to TMZ treatment. # - Indicates a ≥ 2-fold increase in the ratio of LC-3II/LC-3I. Asterisks indicate ≥ 2-fold increases or decreases due to AKT inhibitor IV treatment. t = trend. **B**. Means for surviving fraction ± SD for LN443 cells ± AKT inhibitor IV ± TMZ for 1500 cells/60-mm dish. * p = 0.0075. **C**. Means for surviving fraction ± SD for LN443 cells ± AKT inhibitor IV ± TMZ for 1500 cells/60-mm dish. *, ** p ≤ 0.0003, *** p = 0.03. Plating 3000 cells gave similar results. **D**. Representative Western of LN443 cells untreated or treated with AKT inhibitor IV at the concentrations indicated for 2 days before lysing at days 4 or 6.

It was surprising that a reduction in total AKTs did not impact the level of pAKT. We therefore treated LN443 cells with AKT inhibitor IV to specifically assess the effects of pAKT in the absence or presence of TMZ (Figure 8). These results indicate that suppression of pAKT suppressed SPARC. The suppression of SPARC suggests that unlike total AKT, pAKT regulates SPARC expression in these cells.

Inhibition of AKT activity correlated with decreased caspase 3 cleavage, but increased caspase 7 cleavage. Although suppression of pAKT did not induce PARP cleavage after two days of treatment (Figure 8A), the increase in cleaved caspase 7 by day 2 may contribute to the slight delayed apoptosis observed by days four and six (Figure 8D, and see Additional file 2: Figure S2, Panel F for proposed signaling mechanisms).

As expected, the inhibitor induced autophagy in these cells as indicated by decreased phospho- and total PRAS40 by days 4 and 6 (Figure 8D), the increase in LC3-II by day 2 (Figure 8A) and maintenance of high LC3-II by days 4 and 6 with corresponding decrease on p-p62 (upper band) and increased p62 (lower band) by days 4 and 6 (Figure 8D). The suppression of pAKT and induction of autophagy was accompanied by decreased survival in the absence or presence of TMZ (Figure 8B).

TMZ did not alter the signaling observed with AKT IV (Figure 8A). As the lowest dose of AKT inhibitor IV was sufficient to induce death of all cells in the clonogenic assay (Figure 8B), the ability of AKT inhibitor IV to sensitize cells to TMZ treatment was studied using lower doses of both agents. While increasing AKT inhibitor IV correlated with decreasing surviving fraction, AKT inhibitor IV did not further sensitize cells to TMZ (Figure 8C). The data further demonstrate that 0.125 μM AKT inhibitor IV is more efficient than 80 μM TMZ (Figure 8C).

The combined data for the LN443 cells indicate that HSP27 regulates SPARC and pAKT in these cells, and its suppression is accompanied by decreased survival due to enhanced apoptosis and autophagy. However, targeting SPARC alone is not a good therapeutic approach as tumor cell survival is increased. Interestingly, loss of SPARC due to HSP27 or pAKT inhibition is not detrimental, suggesting that the death signaling induced by HSP27 and pAKT inhibition takes precedence.

In TMZ, the SPARC-induced death signaling is impacted by a reduction in total AKTs, but survival in TMZ is not suppressed and this correlates with the maintenance of pAKT despite a decrease in total AKT. Indeed inhibition of pAKT suppresses survival of cells in the presence of TMZ.

Therefore, we have demonstrated that SPARC, HSP27, and pAKT affect the expression and function of each other. The data also indicate that, whether SPARC expression is independent or dependent on HSP27, HSP27 inhibition is effective in reducing the survival of the cells. However, if SPARC expression is independent of HSP27, pAKT will be high despite the inhibition of HSP27, and the tumor cells will survive better in TMZ.

### Inhibition of HSP27 decreases tumor cell survival in primary glioma cells

These data were established using cell lines having high SPARC expression and similar genetic backgrounds with respect to PTEN and p53 status (see Additional file 4: Table S1). As the majority of gliomas have high SPARC expression, these data suggest that inhibition of HSP27 ± pAKT may be useful therapeutic approaches. To determine whether their inhibition would be useful for primary brain tumors that may have different mutation profiles, we selected two primary GBM-derived cell lines having similar HSP27 and SPARC expression profiles, but which differed in their PTEN, MGMT, and p53 status (see Additional file 4: Table S1).

**Figure 9:**
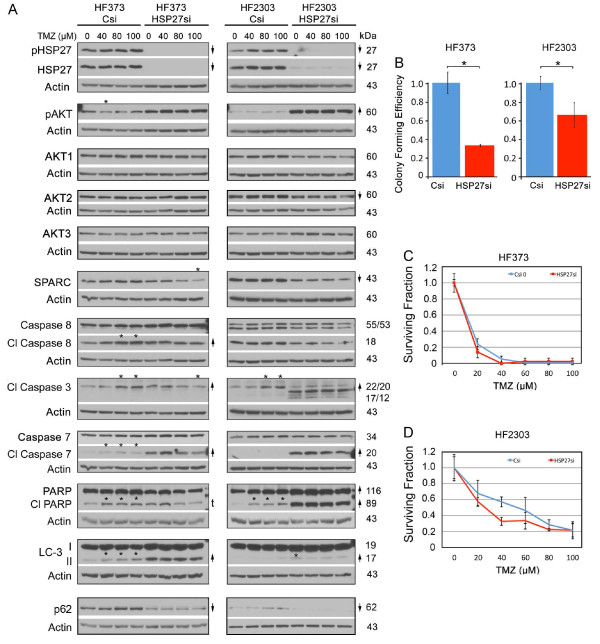
**Inhibition of HSP27 suppresses survival but does not alter sensitivity to temozolomide (TMZ) in primary glioma cell lines**. **A**. Representative Western blots of HF373 and HF2303 cells treated with control siRNA (Csi) or HSP27 siRNA (HSP27si) for 72 hr. Equal numbers of siRNA-treated cells were plated overnight in growth serum and then treated with TMZ at the concentrations indicated for 2 days before lysing. Arrows indicate ≥ 2-fold increases or decreases due to siRNA treatment. # - Indicates a ≥ 2-fold increase in the ratio of LC-3II/LC-3I. Asterisks indicate ≥ 2-fold increases or decreases due to TMZ treatment. t = trend. **B**. Means for colony forming efficiency ± SD for HF373 and HF2303 cells ± HSP27 siRNA for 3000 cells/60-mm dish. HF373 * p = 0.0006; HF2303 * p = 0.017. **C, D**. Means for surviving fraction ± SD for HF373 and HF2303 cells treated with Control (Csi) or HSP27 (HSP27si) ± TMZ for 3000 cells/60-mm dish. Plating 1500 cells gave similar results.

For HF373 tumor cells, HSP27 inhibition did not suppress SPARC or pAKT, suggesting that in this primary cell line, SPARC expression was not under control of HSP27 (Figure 9A). Similar to the H2 SPARC-GFP expressing cells (Figure 3), HSP27 inhibition resulted in increased pro-apoptotic (cleaved caspase 8, caspase 3, caspase 7 and PARP) and pro-autophagic (increased LC3-II and decreased p62) signaling, with maintenance of pAKT levels (Figure 9A). Inhibition of HSP27 correlated with decreased tumor cell survival in the clonogenic assay (Figure 9B).

The HF373 cells are MGMT negative (Additional file 5: Figure S4), and thus are highly susceptible to TMZ treatment (Figure 9C). As expected, TMZ treatment of control siRNA-treated cells was associated with increased pro-death signaling (Figure 9A), which was eliminated by inhibition of HSP27 (Figure 9A), but as also anticipated, HSP27 inhibition did not alter tumor cell survival in TMZ.

In HF2303 tumor cells, inhibition of HSP27 did decrease SPARC expression by 50%, but the decrease in both SPARC and HSP27 was not sufficient to decrease pAKT levels, suggesting additional pathways independently governing pAKT expression in these cells. However, HSP27 inhibition was accompanied by increased pro-apoptotic (cleaved caspase 3, caspase 7 and PARP), but very low pro-autophagic (LC3-II and p62) signaling (Figure 9A), likely due to the inhibition of autophagy by the very high levels of pAKT. The increase in apoptotic signaling combined with the lack of autophagy signaling resulted in greater tumor cell survival in the clonogenic assay (Figure 9B).

The HF2303 cells are MGMT-positive (Additional file 5: Figure S4), and are less responsive to TMZ treatment (Figure 9D). As expected, the maintained high levels of pAKT (Figure 9A) correlated with the inability of HSP27 inhibition to suppress tumor cell survival in TMZ (Figure 9D).

**Figure 10:**
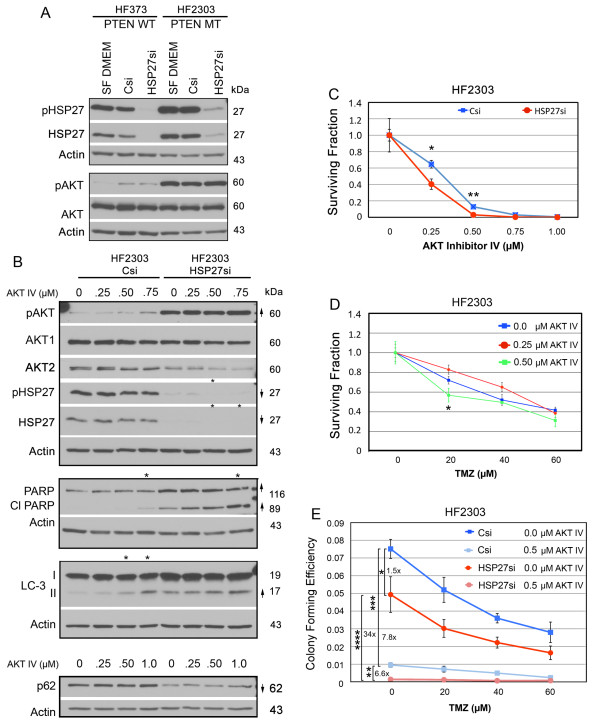
**Inhibition of pAKT augments HSP27 siRNA suppression of survival but does not alter sensitivity to temozolomide (TMZ) in primary glioma cell lines**. **A**. Representative Western blots of HF373 and HF2303 cells grown in serum-free medium (SF DMEM) or treated with control siRNA (Csi) or HSP27 siRNA (HSP27si) for 72 hr. **B**. Representative Western blots of HF2303 cells treated with control siRNA (Csi) or HSP27 siRNA (HSP27si) for 72 hr. Equal numbers of siRNA-treated cells were plated overnight in growth serum then treated ± AKT inhibitor IV at the concentrations indicated for 2 days before lysing. Arrows indicate ≥ 2-fold increases or decreases due to siRNA treatment. # - Indicates a ≥ 2-fold increase in the ratio of LC-3II/LC-3I. Asterisks indicate ≥ 2-fold increases or decreases due to AKT inhibitor IV treatment. **C**. Means for surviving fraction ± SD for HF2303 cells treated with Control (Csi) or HSP27 (HSP27si) siRNAs ± AKT inhibitor IV plating 3000 cells/60-mm dish. * p = 0.0061, ** p = 0.0008. **D**. Means for surviving fraction ± SD for HF203 cells treated ± AKT inhibitor IV ± TMZ plating 3000 cells/60-mm dish. * p = 0.029. **E**. Means for colony forming efficiency ± SD for HF2303 cells treated with Csi or HSP27si siRNAs ± AKT inhibitor IV ± TMZ plating 3000 cells/60-mm dish. * p = 0.017, ** p = 0.0005, *** p = 0.0001, and **** 0.0012. Plating 6000 cells/60-mm dish gave similar results.

These data suggest that in primary human gliomas, unlike LN433 cells, targeting HSP27 alone is not sufficient to suppress SPARC and pAKT, and that other genetic mutations may also play a role. We have previously demonstrated that PTEN is capable of suppressing SPARC-induced pAKT [48]. An examination of the PTEN status of the corresponding primary tumors, available via TCGA website http://cancergenome.nih.gov/, indicated that HF373 is likely PTEN wildtype while HF2303 is PTEN mutant. These data suggest that in HF373 cells, despite high SPARC expression, PTEN, acting downstream of SPARC, can inhibit SPARC-induced signaling to promote higher levels of pAKT. Conversely, the combination of high SPARC and mutant PTEN may super-induce pAKT. A direct comparison of pAKT levels between the two cells lines (Figure 10A) indicates that the pAKT levels are indeed higher in the PTEN-mutant tumor cells. Therefore, in the HF2303 cells, HSP27 inhibition was insufficient to suppress the elevated pAKT, which was likely enhanced by PTEN loss.

### Combined HSP27 inhibition and pAKT suppression decreases tumor cell survival better than either alone

To determine whether inhibition of pAKT could augment suppression of HSP27 as a therapeutic approach, HF2303 cells were treated with control or HSP27 siRNAs in combination with increasing amounts of AKT inhibitor IV. Inhibition of pAKT alone induced increased LC3-II along with a slight increase in cleaved PARP. As with LN443 cells, induction of autophagy was greater at day 4 (data not shown). HSP27 siRNA alone induced apoptosis (cleaved PARP) and autophagy (increased LC3-II and decreased p62) signaling. Indeed, the combination treatment increased PARP cleavage (Figure 10B). The 0.50 μM dose of AKT inhibitor IV alone suppressed tumor cell survival (Figure 10C), and sensitized cells to lower doses of TMZ (20 μM; Figure 10D). Importantly, the combination of HSP27 inhibition plus 0.5 μM AKT inhibitor IV suppressed the surviving fraction more than inhibition of HSP27 alone (Figure 10E), and was more effective than TMZ alone (Figure 10E).

The studies using the primary human glioma cells lines indicate that in cells having high SPARC and HSP27 but low pAKT, targeting HSP27 does suppress survival by inducing apoptotic and autophagic signaling. In cells with high SPARC, HSP27, and pAKT, inhibiting HSP27 suppresses survival primarily through apoptotic signaling, but combined HSP27 inhibition plus inhibition of pAKT better suppresses survival by inducing apoptotic and autophagic signaling.

## Discussion

The current treatment regimen for glioma patients is surgery, followed by RT plus TMZ, followed by 6 months of adjuvant TMZ [7,8]. While this treatment protocol has benefited a subpopulation of GBM patients [9], the overall survival rate for the majority of primary GBM patients is still less than 1 year [10]. Consequently, additional or different therapies are required.

SPARC has been proposed both as a therapeutic target and as a therapeutic agent. It is suggested that the opposing designations may be due to cancer-type or cell-type specific differences. In glioma, we previously demonstrated that SPARC promotes invasion, but suppresses proliferation, suggesting that it may be a therapeutic target for invasion, but conversely, a therapy to inhibit proliferation (29). We also demonstrated that SPARC increases total and pHSP27, which promotes migration and invasion [28]. As SPARC can also increase AKT phosphorylation, and as HSP27 and AKT interact to regulate the activity of each other, we proposed that inhibition of HSP27 may be a therapeutic approach to inhibit both SPARC-induced glioma cell invasion and survival. Therefore, this study was undertaken to determine whether SPARC itself is a therapeutic target or therapy to suppress glioma cell survival, to determine whether HSP27 inhibition is a better therapeutic strategy to suppress SPARC-induced survival, and to determine whether SPARC or HSP27 inhibition sensitizes glioma cells to radiation treatment or TMZ chemotherapy.

Two established cell line models were used, a forced SPARC expression model (U87 cells transfected with control GFP or SPARC-GFP fusion proteins) and an unforced SPARC expression model (LN443 cells). These cell lines are matched for PTEN (mutant) and p53 (wild-type) status. PTEN-mutant cell lines were chosen as we previously demonstrated that PTEN reconstitution suppresses pAKT even in the presence of forced SPARC [48]. Neither upregulating SPARC in U87 cells nor suppressing SPARC in LN443 cells had any effect on tumor cell survival after radiation therapy (Figure 1). Therefore the remainder of the study focused on the mechanisms of SPARC-induced tumor cell survival ± TMZ treatment, and the effects of SPARC, HSP27 or AKT or pAKT inhibition ± TMZ on SPARC-induced survival, using the above established cell lines and primary human glioma cell lines.

Our studies found the following. **1) **SPARC increases the expression of pro-survival and pro-death protein signaling in balance (Figure 3), and, as a net result, tumor cell survival remains unchanged (Figure 2). **2) **Suppressing SPARC increases tumor cell survival, indicating it is not a good therapeutic target (Figure 6). **3) **Suppressing HSP27 decreases tumor cell survival in all gliomas (Figures 2, 5, 9), but is more effective in SPARC-expressing tumor cells due to the removal of HSP27 inhibition of SPARC-induced pro-apoptotic signaling (Figures 3, 5, 9). **4) **Suppressing total AKT1/2 paradoxically enhanced tumor cell survival, indicating that AKT 1 or 2 are not good therapeutic targets (Figure 7). **5) **However, inhibiting pAKT suppresses tumor cell survival (Figures 4, 8). **6) **Inhibiting both HSP27 and pAKT synergistically decreases tumor cell survival (Figure 10). **7) **There appears to be a complex feedback system between SPARC, HSP27, and AKT (Figures 3, 5, 6). **8) **This interaction is likely influenced by PTEN status (Figure 10).

With respect to chemosensitization, we found the following. **1) **SPARC does enhance pro-apoptotic signaling in TMZ (Figure 3). **2) **Despite this enhanced signaling, SPARC protects cells against TMZ (Figures 2). **3) **This protection can be reduced by inhibiting pAKT (Figure 4). **4) **Combined inhibition of HSP27 and pAKT is more effective than TMZ treatment alone (Figure 10).

Our results shed some insight into the seemingly disparate reports on the function of SPARC as a therapeutic agent versus a therapeutic target. As noted, SPARC increases invasion of glioma cells, but also has a suppressive effect on their growth. This raised concerns that the inhibition of SPARC itself would not be a suitable therapeutic target, as suppression could lead to increased proliferation. Indeed, our studies show that inhibition of SPARC leads to increased tumor cell survival. The mechanism for this is unknown, but may relate to its ability to suppress cell cycle progression [15] and the alleviation of this repression.

Looking at downstream SPARC-induced signaling pathways, we surprisingly found that SPARC upregulates both pro-survival and pro-death signaling proteins. Indeed, independent analysis of one signaling pathway versus the other would lead to different conclusions regarding the use of SPARC as therapy or target. It was interesting to find that the pro-survival signals directly impede the pro-death signaling pathways (45). Therefore, the final effect is that SPARC expression itself does not alter the overall tumor cell survival. However, inhibition of downstream survival signaling proteins HSP27 or pAKT undermines SPARC-induced survival signaling, shifting the balance to increased death signaling. As result, SPARC would be advantageous when suppressing tumor cell survival with HSP27 or pAKT inhibition.

The data suggest a complex interaction and/or feedback system regarding these three proteins. SPARC can upregulate HSP27 and pAKT. Inhibition of HSP27 suppresses pAKT and SPARC expression, and inhibition of pAKT can suppress SPARC. In all cell lines examined though, inhibition of HSP27 decreased survival.

It was surprising that HSP27 depletion could simultaneously decrease total AKT and increase pAKT levels. This was not an artifact due to the inability to strip the pAKT antibody from the membrane. In addition, total AKT2 and AKT3 were probed independently of pAKT, and total AKT2 was decreased. Therefore, the data suggest that there is an unknown mechanism for decreasing total AKT while maintaining high pAKT. Possibly this is due to depletion of a specific AKT isoform, while phosphorylation of the remaining isoform(s) is detected. Interestingly, this inverse relationship between total and pAKT has been described in COS-7 cells undergoing heat shock [49].

In addition, the extent of SPARC and pAKT suppression by HSP27 depletion is likely influenced by other factors that can independently regulate pAKT, such as the genetic background of the tumor cells with respect to PTEN status. It is well established that the loss of PTEN expression correlates with increased pAKT and decreased patient survival [50,51]. Consequently, some gliomas may need to be targeted not only to suppress HSP27 but also to suppress pAKT.

Using AKT as a therapeutic target has been a focus of study for many cancers [52] including gliomas [53] due to its pivotal role in regulating apoptosis and autophagy [54,55]; however, its use is complicated by the fact that there are three AKT isoforms (AKT1, AKT2, AKT3), which are functionally distinct despite sharing a great deal of sequence homology [56-59]. In addition, their functions may differ in a cell-type specific manner. With respect to brain tumors, the level of AKT1 found in glioma cells and tissues was more similar to that found in normal human astrocytes or non-neoplastic regions of the brain [60]; whereas AKT2 levels were increased [60,61], and AKT3 levels were decreased [60]. Interestingly, AKT2 has been associated with suppression of apoptosis and increased invasion, as blocking AKT2 induced apoptosis and decreased MMP2 and MMP9 [61,62].

In our cells, siRNA inhibition of AKT1/2 was partial and resulted in increased tumor cell survival, while suppression of AKT3 had no impact on tumor cell survival in the clonogenic assay. Neither of the siRNA treatments greatly affected apoptosis or autophagy signaling, and this may be due to the inability to completely suppress pAKT using this approach. When cells were treated with an inhibitor of AKT, autophagy signaling was significantly increased and tumor cell survival was significantly decreased. These results emphasize the need to assess effects not only on total AKT but also pAKT. Our results suggest that the maintenance of pAKT in the face of decreased total AKT could actually promote tumor cell survival.

In our studies we used AKT inhibitor IV, which blocks activation of AKT downstream of PI-3 K [53]. However, the different cell lines were differentially sensitive to the inhibitor. When SPARC expression is forced, the cells require higher concentrations of inhibitor to suppress pAKT. In contrast, when SPARC is not forced, inhibition of AKT suppressed pAKT and total AKT2. While the suppression of pAKT was associated with increased autophagic signaling, it had no affect on apoptosis. Interestingly, inhibition of pAKT decreased SPARC and caspase-3, supporting the contention that AKT can regulate SPARC expression.

The literature suggests that SPARC is a chemosensitizer [22-24]. We therefore investigated the effects of SPARC with respect to TMZ as it is the current chemotherapy for glioma patients. We used concentrations of TMZ up to 100 μM, which corresponds to serum levels achieved in patients for treatment of gliomas [63]. The drug induces autophagy [47] and apoptosis [46,64]. However, the drug does not induce apoptosis in U87 cells after three days [46], but does so after prolonged incubations of six [46] or eight days [64]. LN443 cells are quite resistant, even at 100 μM for eight days [64], and this may be because the drug is less effective at inducing apoptosis in p53 wild-type glioma cells [64]. Therefore, to determine whether SPARC expression or siRNA inhibition of HSP27 or AKTs enhanced the sensitivity of these PTEN-mutant, p53 wild-type glioma cells to TMZ, we treated the cells for 48 hr when no effects from TMZ alone are observed.

When the SPARC-expressing glioma cells were treated with TMZ, our results are consistent with previous reports that suggest SPARC is a therapeutic agent. We observed a SPARC-induced increase in procaspase 8, cleaved caspase 8, cleaved caspase 7, and cleaved PARP in the presence of TMZ. It is interesting to note that caspase 8-induced apoptosis can be inhibited by procaspase 8/AKT/integrin beta 1 complex [65]. However, SPARC may interfere with this anti-apoptotic complex either by disrupting cell surface adhesion outside the cell, and/or by binding to and activating procaspase 8 within the cell [23]. Activation of caspase 8 can activate caspase 7, which in turn can cleave PARP [66,67]. These data support our observations that SPARC siRNA resulted in the loss of TMZ-associated death signaling which was accompanied by decreased procaspase 8, cleaved caspase 7 and cleaved PARP. Additionally AKT 1/2/3 inhibition served to decrease SPARC-induced death signaling in TMZ. These combined observations suggest that the mechanism involved is not chemotherapy-specific, but SPARC-specific. However, despite neither siRNA treatment resulting in the loss of this signaling, inhibition of this pathway did not have a big impact on tumor cell survival, also supporting the conclusion that SPARC is not a strong chemosensitizer.

In fact, the data suggest that despite SPARC-induced death signaling, SPARC actually protects cells against TMZ treatment. Furthermore, the data show that pAKT mediates this effect, as AKT inhibition using AKT inhibitor IV removes the survival advantage. HSP27 inhibition was also shown to suppress pAKT depending on the cell line examined and whether SPARC expression was forced or not. The data, however, suggest that other genetic changes might influence the status of pAKT, rendering it unregulatable by HSP27. For example, the loss of PTEN, a common aberration in gliomas, results in elevated pAKT. It is less clear as to whether PTEN status influences the outcome of TMZ treatment, as there are reports that wildtype PTEN correlates with increased survival in the drug-treated patients [68-70] and those demonstrating no benefit [71]. Additionally, the outcome might be influenced by MGMT promoter methylation status. Patients having tumors with PTEN-positive tumors with methylated MGMT had a survival advantage when treated with TMZ plus erlotinib and RT [72]. Further studies are needed.

The combined data using established cell lines indicate that blocking HSP27 is an effective treatment approach, but is more effective if SPARC expression is forced (not regulated by HSP27) and promotes death signaling. A major question is whether primary gliomas have forced or non-forced SPARC expression. Two primary human glioma cell lines were treated with control or HSP27 siRNA in the absence or presence of TMZ. Treatment with HSP27 siRNA resulted in decreased colony forming efficiency for both cell lines (Figure 9B). However, the effects of HSP27 inhibition on signaling were different between the cell lines (Figure 9A).

Inhibition of HSP27 in HF373 cells did not eliminate SPARC suggesting a "forced SPARC" expression profile as observed for the H2 cells. In contrast to the H2 cells where high SPARC expression correlated with high pAKT, pAKT remained low in the HF373 cells. As we had previously demonstrated that PTEN reconstitution could suppress SPARC-induced activation of AKT [48], we considered the PTEN status for this cell line. No mutation has yet been described for HF373, suggesting a wildtype status. This suggests that wildtype PTEN suppresses SPARC-induced pAKT in these cells.

For HF2303 cells, inhibition of HSP27 only reduced SPARC by 50% and pAKT remained high, also suggesting a forced SPARC profile. In addition, PTEN is mutant in HF2303. Therefore, SPARC expression combined with loss of PTEN was sufficient to promote elevated pAKT. Thus, the two cell lines had a "forced" SPARC expression profile, but the resultant effect on pAKT levels differed, likely due to differences in the PTEN status.

As a consequence the loss of HSP27 promoted apoptotic signaling in both cell lines. However, the HF373 cells (low pAKT) demonstrated increased autophagy, whereas the HF2303 cells (high pAKT) did not. In the latter cells, autophagy was induced with the AKT IV inhibitor. These observations are in agreement with observations that knockdown of AKT activity increases autophagy, and apoptosis is not the prevailing response [73]. Thus, we propose that HSP27 inhibition alone will be most effective in SPARC-positive/PTEN-wildtype tumors, while combined inhibition of HSP27 and pAKT will likely be warranted for tumors that are SPARC-positive/PTEN-null. Furthermore, this treatment is more effective than treating with TMZ alone. As recent reports indicate that TMZ and RT can produce a hypermutation phenotype, affecting up to 30% of patients [74], a treatment regimen that eliminates TMZ may be highly advantageous. As HSP27 and AKT are already the targets of clinical trials, the rationale for their use has been established. Furthermore, inhibition of pHSP27 and/or AKT as a therapeutic approach has been proposed for prostate and bladder cancer [75-77]. Studies are therefore initiated to determine whether the strategies demonstrated here will be successful *in vivo *to treat gliomas.

## Conclusions

We conclude that inhibition of HSP27 alone, or in combination with pAKT inhibitor IV, may be valuable therapeutic approaches to inhibit SPARC-induced glioma cell invasion and survival in SPARC-positive/PTEN-wildtype or SPARC-positive/PTEN-null tumors, respectively.

## Methods

### Materials

Standard reagents were purchased from Fisher Scientific (Hanover Park, IL) or VWR (West Chester, PA); others as follow. Invitrogen Life Technologies (Carlsbad, CA): Standard tissue culture reagents. ATCC (Manassas, VA): U87MG and LN443 cells. Millipore (Bedford, MA): Immobilon P membranes. Thermo Fisher (Rockford, IL): ECL chemiluminescence kit. Denville Scientific (Metuchen, NJ): High Blot CL autoradiography film. BioRad: nonfat dry milk. Sigma-Aldrich (St. Louis, MO): DMSO. LKT Laboratories, Inc. (St. Paul, MN): temozolomide. Calbiochem/EMD Biosciences (San Diego, CA): AKT Inhibitor IV (124011). **siRNAs: **Dharmacon (Lafayette, CO): control siRNA (D001210-01), and human HSP27 siRNA (HSPB1 L-005269-00-0010). Santa Cruz Biotechnology (Santa Cruz, CA) SPARC siRNA (sc-37166), AKT1/2 siRNA (sc-43609), and AKT3 siRNA (sc-38911). **Antibodies: **Haematologic Technologies (Essex Junction, VT): SPARC (AON-5031). Cell Signaling Technology (Danvers, MA): phospho-HSP27 (Ser82; 2401), phospho-AKT (Ser473; 4501), AKT (9272), AKT1 (2938), AKT2 (2964), p62 (5114), caspase 7 (9492), cleaved caspase 7 (9491), and PARP (9542). ImGenex (San Diego, CA): caspase-8 (IMG-224A), and caspase-3 (IMG-144A and IMG145). Sigma-Aldrich (St. Louis, MO): LC-3 (L7543). Santa Cruz Biotechnology (Santa Cruz, CA): Goat anti-rabbit HRP secondary antibody, goat anti-mouse HRP, donkey anti-goat HRP, HSP27 (sc-1049), Beclin 1 (sc-11427), actin (sc-1616) and GAPDH (sc-20357). Millipore (Temecula, CA): AKT3 (07-383), pPRAS40 (07-1282), and PRAS40 (05-1070). BD Pharmingen (San Diego, CA): MGMT (557045).

### Cell lines and cell maintenance

The generation of the SPARC-GFP (H2) and control GFP (C1.1) clones was previously described [28]. Cells were maintained in DMEM + 10% FBS and geneticin (400 μg/ml). LN443 was maintained in DMEM + 5% FBS. Primary human cells were maintained in DMEM + 10% FBS. All cells are maintained in 1% penicillin:streptomycin (1:1). A summary of the cell lines used is presented in Additional file 4: Table S1.

### Imaging

An Olympus 1 × 50 fluorescence microscope attached to an Insight SPOT 4 camera was used to capture images at × 40 magnification using SPOT software. Composite Western images were prepared using Photoshop CS3 software.

### Clonogenicity assays

Cells were trypsinized, counted with a hemocytometer, and plated in triplicate at 375, 750, 1,000, 1500, 3,000 or 6,000 cells/60-mm tissue culture dish (as indicated in figure legends), with media changes every 3-4 days. After ~10 days for each experiment, colonies were washed once with PBS, and then fixed in 100% methanol for 20 min at -20°C. The cells were rinsed twice with PBS, stained in 10% Giemsa for 10-15 min, and then rinsed clean in distilled water. After drying, the stained colonies having at least 50 cells were counted by at least 2 individuals. The colony-forming efficiency (CFE) was calculated as the number of colonies/number of cells plated. The surviving fraction (SF) was calculated as the number of colonies/[(number of cells plated) (CFE of the control untreated cells)]. Representative assays are illustrated for an n = 2 (RT) or n = 2 or 3 (± TMZ ± siRNA) experiments.

For RT survival curves, the cells were plated as above, allowed to attach for 24 hr, and then irradiated with 1-5 or 10 Gy. The control dishes were unexposed to radiation, but otherwise handled the same. Radiation exposure of cell cultures was performed using a 5000 Ci Cesium (Cs-137) irradiator (Mark I; J. L. Shepherd and Associates, San Fernando, CA). The next day, media were changed, and the colonies were allowed to develop as above.

For TMZ treatment, cells were plated as above, allowed to attach for 24 hr, and then treated with 0 (0.1% DMSO), 10, 20, 40, 60, 80, or 100 μM TMZ for 2 days. The media were then changed and the colonies were allowed to develop as above.

For experiments incorporating control, HSP27, SPARC, or AKT siRNAs, duplicate 60-mm dishes were plated. After assessing the effectiveness of control and gene-specific siRNA oligos, the oligos for HSP27 (240 nM), SPARC (120 nM), AKT1/2 (12.5 nM), or (AKT3 12.5 nM) were added for 72 hr. Cells were then trypsinized and seeded into 60-mm dishes for the clonogenic assay or 6-well plates for Western blot analyses. Cells attached overnight, and were then treated with TMZ. The drug was then removed, the cells rinsed, and fresh media was added. Colonies were allowed to develop as above. For experiments using AKT inhibitor IV, cells were seeded into 60-mm dishes overnight, treated with TMZ (40, 80 or 100 μM), AKT inhibitor IV (0.0625 - 1.0 μM), or both for 2, 4, 6, or 8 days for Western blot analyses (see below). Controls were treated with 0.1% DMSO for TMZ treatments or 0.01% DMSO for AKT inhibitor IV treatments.

### Dye exclusion assay

Dye exclusion assays were performed to ensure that equal numbers of viable cells were plated.

### Western blot analyses

To determine the effects of SPARC expression and siRNA and/or AKT inhibitor IV ± TMZ treatment, cells were plated (6-well plates) for protein lysates, as previously reported [23]. Protein concentration was determined using the BCA protein assay kit (Pierce, Rockford, IL). Five to 25 μg of protein and 5-10 μl of molecular weight marker were subjected to electrophoresis through 8%, 12.5% or 15% SDS-polyacrylamide Tris-glycine gels and were transferred onto Immobilon P membranes. Proteins were detected as previously reported [23]. The primary antibodies were diluted 1:500 for caspase 3, caspase 8 and MGMT; 1:1000 for HSP27, pHSP27, AKT, AKT1, AKT2, AKT3, pAKT, PARP, caspase 7, cleaved caspase 7, LC-3, p62, pRAS40, PRAS40, Beclin1, GAPDH; 1:6500 for SPARC, or 1:2000 for actin. Quantitation was performed using ImageJ software as previously reported [23]. Representative Western blots are illustrated for n ≥ 3 experiments.

### Data and statistical analyses

Two-fold changes in protein levels were considered significant, and the changes are indicated by asterisks (2-fold increase or decrease due to TMZ treatment) or arrows (2-fold increase or decrease due to SPARC expression, or siRNA or AKT inhibitor IV treatment) in the figures. For all statistical analyses the Student's t-test was performed. Statistical significance alpha was set at p ≤ 0.05.

## Abbreviations

RT: Radiation therapy; TMZ: Temozolomide; SPARC: Secreted protein acidic and rich in cysteine.

## Competing interests

The authors declare that they have no competing interests.

## Authors' contributions

CRS performed radiation and clonogenic assays, AKT Inhibitor IV assays, Western blot analyses, and participated in experimental design. WAG performed siRNA inhibition experiments, clonogenic assays, and Western blot analyses. DK performed clonogenic assays. SLB participated in experimental design and execution of radiation assays, and interpreted radiation data. CB critically reviewed data and manuscript. SAR conceived of the overall study and design, performed Image J analyses, analyzed and interpreted all data, wrote and submitted the manuscript. All authors read and approved the final manuscript.

## Supplementary Material

Additional file 1 **Figure S1**. **Timing study of TMZ-induced death in U87 cells**. C1.1 GFP-expressing cells (1 × 10^4^) were plated overnight in 6-well plates. Cells were treated with 0 (0.1% DMSO) or 100 μM TMZ for 2 days, and the media were changed every 2 days. Lysates were harvested on days 6 and day 8. Western blots were probed as indicated. Click here for file

Additional file 2 **Figure S2**. **Proposed mechanisms**. For all panels, solid lines represent SPARC-induced pro-survival and pro-death signaling, dashed lines represent siRNA or AKT inhibitor IV inhibition of signaling, and changes in protein levels due to siRNA or AKT inhibitor IV inhibition of signaling are indicated by changes in color intensity. **Panel A. Proposed mechanisms of forced SPARC-induced signaling in U87 glioma cells alone or treated with temozolomide (TMZ)**. In the absence of TMZ, SPARC promotes migration via upregulation of the p38MAPK-MAPKAPK2-HSP27 signaling pathway [28]. SPARC promotes pro-survival signaling (pHSP27, pAKT) and pro-apoptotic signaling (pro- and cleaved caspase 8 and cleaved caspase 3). Note: pAKT indirectly inhibits cleavage of caspase-3 through inhibition of caspase-9 activation. The dashed blue lines illustrate caspase activation inhibited by HSP27. We propose that the pro-survival and pro-apoptotic signaling cascades balance one another, and cells survive. However, TMZ treatment of SPARC-expressing cells induces caspase 7 and PARP activation as indicated by the green line. Consistent with the literature [23], we propose that integrin beta 1 recruits procaspase 8 and AKT. SPARC binds to procaspase 8 to induce chemosensitivity to TMZ. **Panel B. Inhibition of HSP27 shifts the balance towards SPARC-induced pro-apoptotic signaling**. Dashed blue lines indicate that the loss of HSP27 expression decreases migration and suppresses pro-survival signaling (AKT1, AKT2). The dashed red lines indicate the resultant increase in pro-apoptotic signaling (cleaved caspase 3, 7 and PARP), due to loss of HSP27, resulting in increased apoptosis. Decreased AKT1/2 expression is accompanied by the loss of SPARC-induced sensitivity to TMZ, indicated by the dashed green line. **Panel C. Proposed mechanism of endogenous SPARC-regulated signaling in LN443 glioma cells treated with HSP27 siRNA alone or with temozolomide (TMZ)**. SPARC signaling is proposed to be the same as in U87 cells (Panel A). However, in the absence of forced SPARC expression, HSP27 inhibition suppresses SPARC and pAKT, thereby suppressing SPARC-induced pro-survival and pro-apoptotic signaling, as indicated by the dashed red and blue lines. The loss of HSP27 inhibition permits basal apoptotic signaling through caspase 3, 7 and PARP cleavage. The loss of HSP27 is proposed to decrease migration. In TMZ, the HSP27 inhibition suppresses SPARC-induced sensitivity through caspase 7 and PARP activation, as indicated by the dashed green line. **Panel D. Proposed signaling in LN443 glioma treated with SPARC siRNA alone or with TMZ**. As expected, loss of SPARC eliminates enhanced pro-survival signaling through HSP27 and pAKT and eliminates pro-apoptotic signaling through caspases 8 and 3. Survival is maintained through basal HSP27 and AKT signaling to inhibit apoptosis, as indicated by the red lines. In TMZ, loss of SPARC eliminates caspase 7and PARP cleavage, indicated by the dashed green line. **Panel E. Proposed signaling in LN443 glioma cells treated with AKT1/2/3 siRNA and TMZ**. AKT1/2 or AKT3 inhibition results in decreased total AKT1/2 or AKT3, but not pAKT. We propose that SPARC-induced procaspase 8 expression and cleavage are not affected, permitting the cleavage of caspase 3. However, SPARC-induced upregulation of HSP27 inhibits further cleavage of caspases 3, 8 and PARP, as indicated by the red lines. In TMZ, suppression of either AKT1/2 or AKT3 reduced SPARC-induced cleavage of caspase 7 and PARP, as indicated by the dashed green line. **Panel F. Proposed signaling in LN443 glioma cells treated with AKT inhibitor IV and TMZ**. AKT inhibitor IV suppresses total AKT2, pAKT, SPARC. Loss of pAKT and SPARC permits basal cleavage of caspase 3, but basal levels of HSP27 inhibits further caspase 3 and 8 and PARP cleavage. Unexpectedly, the inhibitor increased cleaved caspase 7 by in the absence or presence of TMZ, but this did not enhance apoptosis. Symbols are courtesy of SABiosciences. Click here for file

Additional file 3 **Figure S3**. **SPARC expression does not protect LN443 cells against temozolomide (TMZ)**. Average surviving fraction ± SD of LN443 cells in 0, 1, 50, and 100 μM TMZ plating 375 cells/60-mm dish. Click here for file

Additional file 4 **Table S1**. **Summary of cell Lines**. Click here for file

Additional file 5 **Figure S4**. **MGMT expression profile**. Westem blot analysis of MGMT protein in glioma lysates. X- empty lane. T98G is a positive control for MGMT. Click here for file
