# Supplementary figures and images for: Inhibition of HSP27 alone or in combination with pAKT inhibition as therapeutic approaches to target SPARC-induced glioma cell survival
Source: Mol Cancer. 2012 Apr 5;11:20. doi: 10.1186/1476-4598-11-20 (PMC3349587; doi:10.1186/1476-4598-11-20)

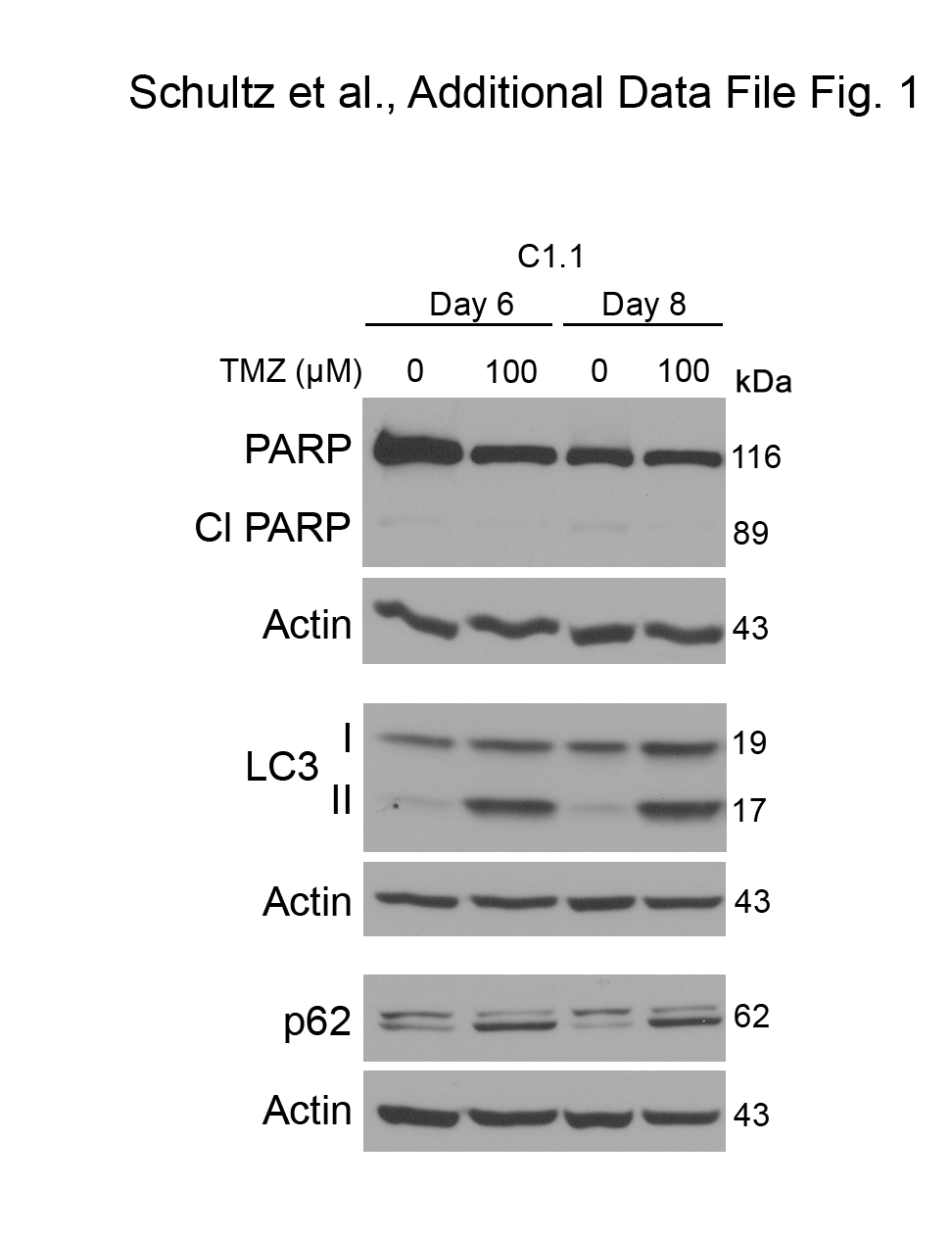

Supplement: Additional file 1 — Figure S1. Timing study of TMZ-induced death in U87 cells. C1.1 GFP-expressing cells (1 × 10^4^) were plated overnight in 6-well plates. Cells were treated with 0 (0.1% DMSO) or 100 μM TMZ for 2 days, and the media were changed every 2 days. Lysates were harvested on days 6 and day 8. Western blots were probed as indicated. [file 1476-4598-11-20-S1.TIFF]

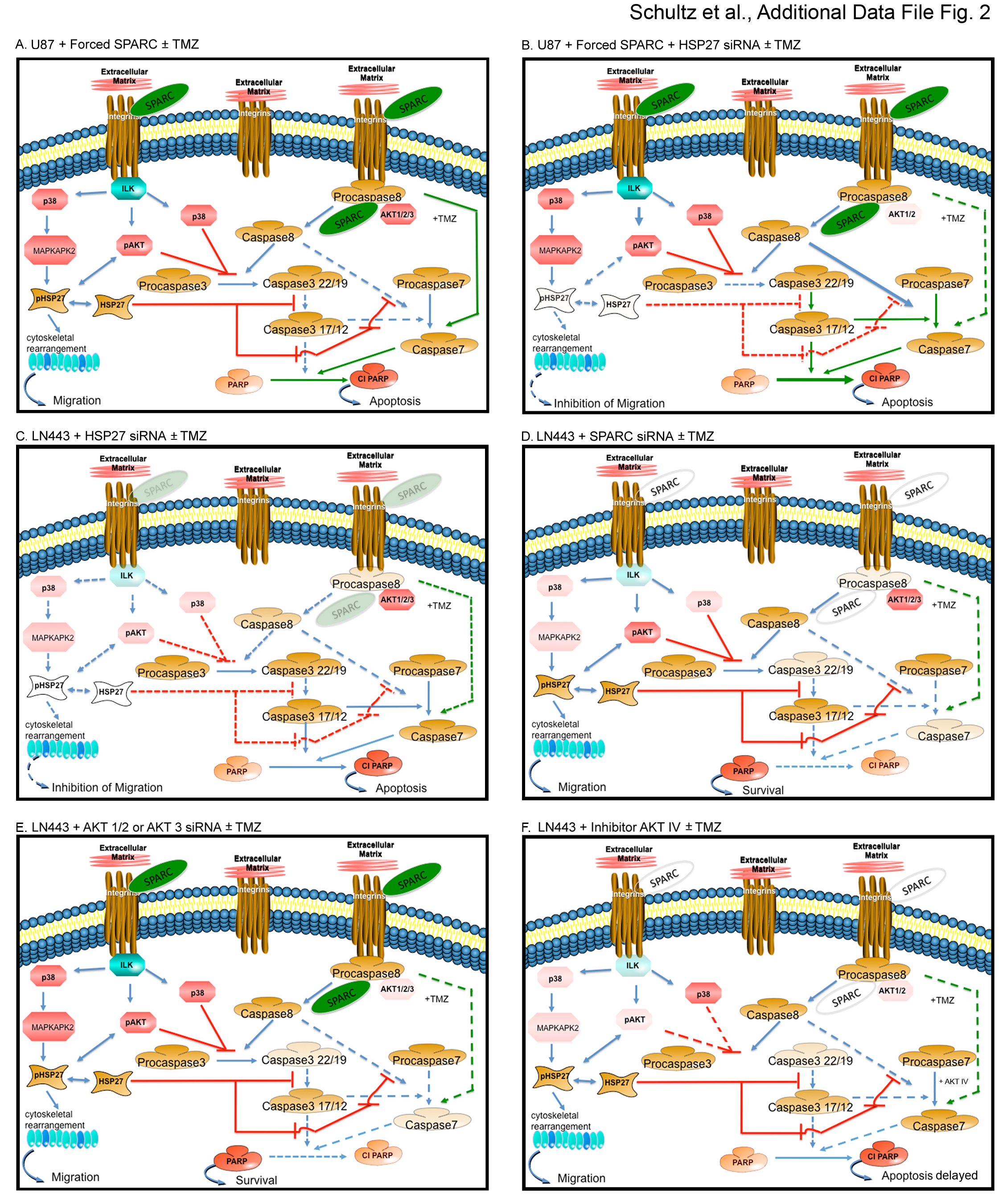

Supplement: Additional file 2 — Figure S2. Proposed mechanisms. For all panels, solid lines represent SPARC-induced pro-survival and pro-death signaling, dashed lines represent siRNA or AKT inhibitor IV inhibition of signaling, and changes in protein levels due to siRNA or AKT inhibitor IV inhibition of signaling are indicated by changes in color intensity. Panel A. Proposed mechanisms of forced SPARC-induced signaling in U87 glioma cells alone or treated with temozolomide (TMZ). In the absence of TMZ, SPARC promotes migration via upregulation of the p38MAPK-MAPKAPK2-HSP27 signaling pathway [28]. SPARC promotes pro-survival signaling (pHSP27, pAKT) and pro-apoptotic signaling (pro- and cleaved caspase 8 and cleaved caspase 3). Note: pAKT indirectly inhibits cleavage of caspase-3 through inhibition of caspase-9 activation. The dashed blue lines illustrate caspase activation inhibited by HSP27. We propose that the pro-survival and pro-apoptotic signaling cascades balance one another, and cells survive. However, TMZ treatment of SPARC-expressing cells induces caspase 7 and PARP activation as indicated by the green line. Consistent with the literature [23], we propose that integrin beta 1 recruits procaspase 8 and AKT. SPARC binds to procaspase 8 to induce chemosensitivity to TMZ. Panel B. Inhibition of HSP27 shifts the balance towards SPARC-induced pro-apoptotic signaling. Dashed blue lines indicate that the loss of HSP27 expression decreases migration and suppresses pro-survival signaling (AKT1, AKT2). The dashed red lines indicate the resultant increase in pro-apoptotic signaling (cleaved caspase 3, 7 and PARP), due to loss of HSP27, resulting in increased apoptosis. Decreased AKT1/2 expression is accompanied by the loss of SPARC-induced sensitivity to TMZ, indicated by the dashed green line. Panel C. Proposed mechanism of endogenous SPARC-regulated signaling in LN443 glioma cells treated with HSP27 siRNA alone or with temozolomide (TMZ). SPARC signaling is proposed to be the same as in U87 cells ( [file 1476-4598-11-20-S2.TIFF]

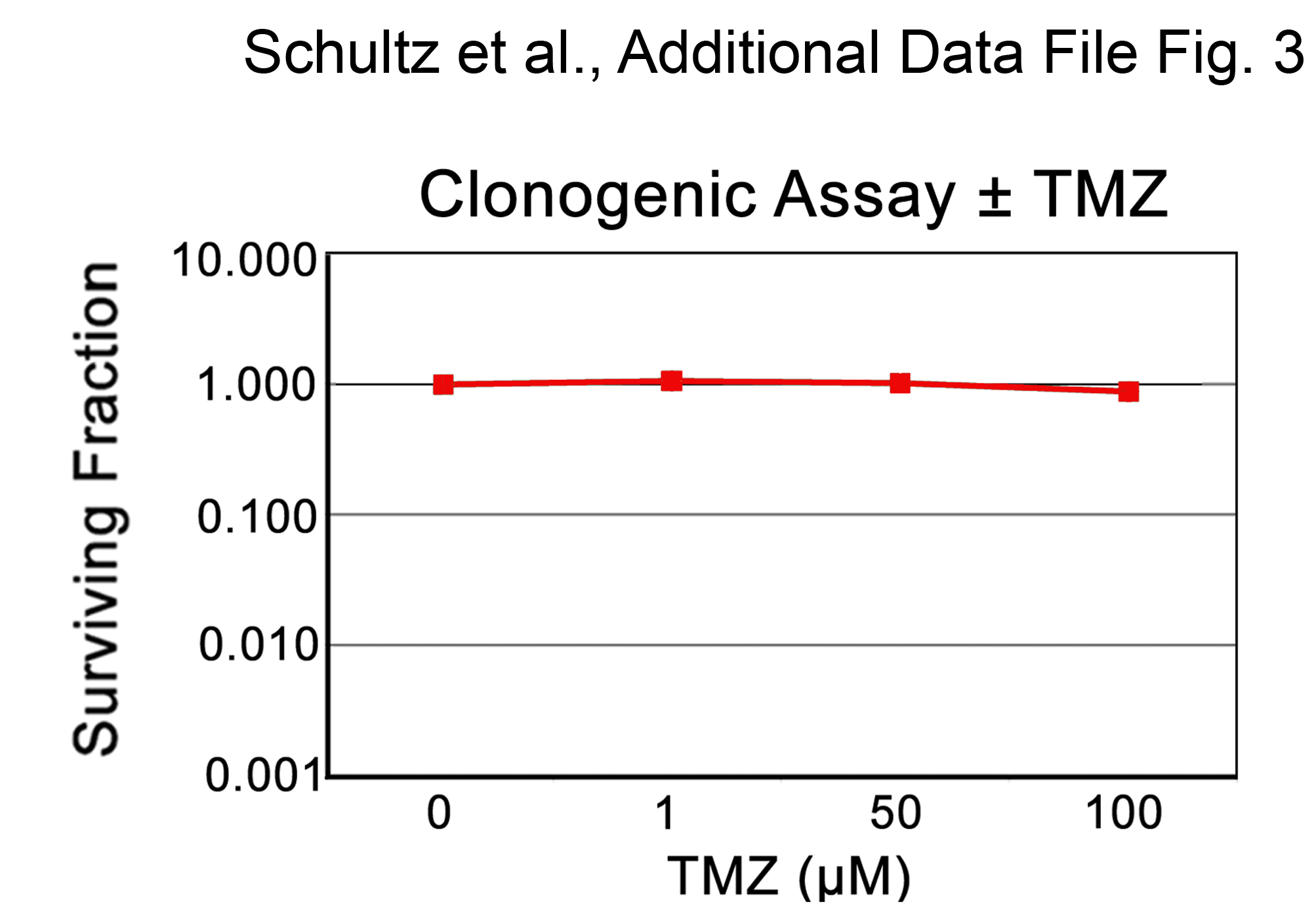

Supplement: Additional file 3 — Figure S3. SPARC expression does not protect LN443 cells against temozolomide (TMZ). Average surviving fraction ± SD of LN443 cells in 0, 1, 50, and 100 μM TMZ plating 375 cells/60-mm dish. [file 1476-4598-11-20-S3.TIFF]

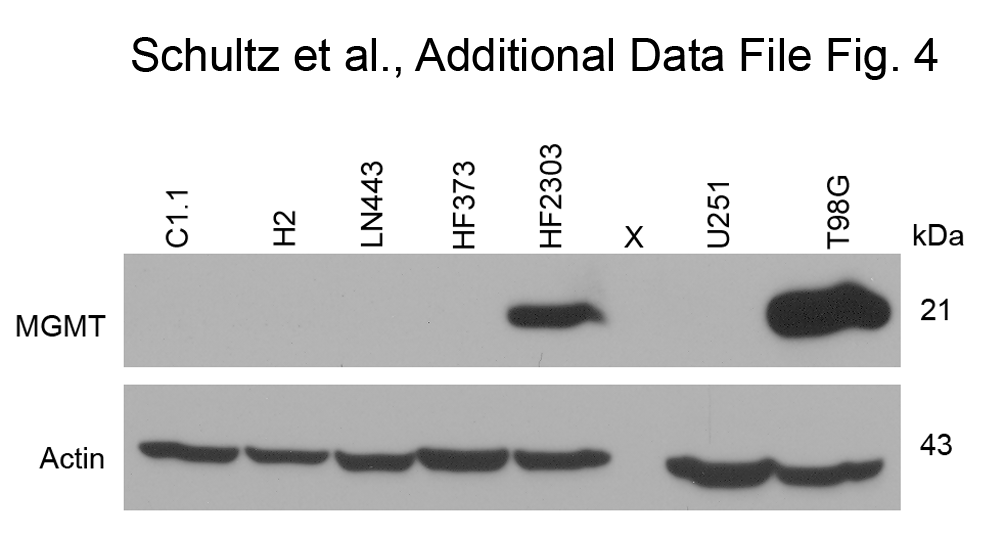

Supplement: Additional file 5 — Figure S4. MGMT expression profile. Westem blot analysis of MGMT protein in glioma lysates. X- empty lane. T98G is a positive control for MGMT. [file 1476-4598-11-20-S5.TIFF]
