# Supplementary material for: Inhibition of HSP27 alone or in combination with pAKT inhibition as therapeutic approaches to target SPARC-induced glioma cell survival
Source: Mol Cancer. 2012 Apr 5;11:20. doi: 10.1186/1476-4598-11-20 (PMC3349587; doi:10.1186/1476-4598-11-20)
Supplement: Additional file 4 — Table S1. Summary of cell Lines. [file 1476-4598-11-20-S4.DOC]

Schultz et al., Additional Data File Table 1

| **Table 1. Summary of Cell Lines** | | | | | |
| --- | --- | --- | --- | --- | --- |
| **Cell Line** | **C1.1** | **H2** | **LN443** | **HF373** | **HF2303** |
|  | U87-derived | U87-derived |  | Primary GBM | Primary GBM |
| **Protein** |  |  |  |  |  |
| SPARC-GFP |  | Positive |  |  |  |
| GFP | Positive |  |  |  |  |
| Endogenous SPARC | Low | Low | Positive | Positive | Positive |
| HSP27 | Low | Positive | Positive | Positive | Positive |
| P53 | WT | WT | WT | Unknown | Mutant |
| PTEN | Mutant | Mutant | Mutant | Wild-type | Mutant |
| MGMT | Negative | Negative | Negative | Negative | Positive |
